# A Novel PBM for Nanomilling of Drugs in a Recirculating Wet Stirred Media Mill: Impacts of Batch Size, Flow Rate, and Back-Mixing

**DOI:** 10.3390/pharmaceutics16030353

**Published:** 2024-03-02

**Authors:** Hamidreza Heidari, Nontawat Muanpaopong, Gulenay Guner, Helen F. Yao, Donald J. Clancy, Ecevit Bilgili

**Affiliations:** 1 Otto H. York Department of Chemical and Materials Engineering, New Jersey Institute of Technology, Newark, NJ 07102, USA; hh44@njit.edu (H.H.); nm632@njit.edu (N.M.); 2 Drug Product Development, GlaxoSmithKline, Collegeville, PA 19426, USA; gulenay.x.guner@gsk.com (G.G.); helen.f.yao@gsk.com (H.F.Y.); donald.j.clancy@gsk.com (D.J.C.)

**Keywords:** wet stirred media milling, population balance model, drug nanoparticles, breakage kinetics, process development, imperfect mixing

## Abstract

We examined the evolution of fenofibrate (FNB, drug) particle size distribution (PSD) during the production of nanosuspensions via wet stirred media milling (WSMM) with a cell-based population balance model (PBM). Our objective was to elucidate the potential impacts of batch size, suspension volumetric flow rate, and imperfect mixing in a recirculating WSMM. Various specific breakage rate functions were fitted to experimental PSD data at baseline conditions assuming perfect mixing. Then, the best function was used to simulate the PSD evolution at various batch sizes and flow rates to validate the model. A novel function, which is a product of power–law and logistic functions, fitted the evolution the best, signifying the existence of a transition particle size commensurate with a grinding limit. Although larger batches yielded coarser and wider PSDs, the suspensions had identical PSDs when milled for the same effective milling time. The flow rate had an insignificant influence on the PSD. Furthermore, the imperfect mixing in the mill chamber was simulated by considering more than one cell and different back-mixing flow ratios. The effects were weak and restricted to the first few turnovers. These insights contribute to our understanding of recirculating WSMM, providing valuable guidance for process development.

## 1. Introduction

The enhancement of bioavailability stands as an important focus area in pharmaceutical research and development, dedicated to improving the therapeutic effectiveness of drug formulations. Nanoparticles have emerged as highly promising vehicles in this pursuit because of their large surface area, enhanced saturation solubility, sustained release, and targeted delivery to enhance bioavailability [1,2,3]. Nanoparticles, which are referred to here as crystalline particles with diameters between 50 and 500 nm, have a specific surface area that is about two orders of magnitude higher than that of coarse micro-sized crystals. This greater surface area, in conjunction with the higher overall solute mass transfer coefficient resulting from thinner diffusion layer in fluids and the increased saturation solubility, especially of <100 nm particles, enable faster drug dissolution [4].

Wet stirred media milling (WSMM) is the most widely used process in pharmaceutical engineering for producing stable drug nanosuspensions [5], which provide drug bioavailability at higher drug doses per injection volume [5,6]. The wide use of WSMM has originated from its robust, reproducible, scalable, solvent-free, and environmentally friendly operation [2,7,8]. A typical pharmaceutical WSMM process runs in recirculation mode [6], wherein a drug suspension is recirculated between a holding tank and a milling chamber multiple times. High-speed rotation of a stirrer (rotor) induces turbulent motion, leading to repeated stressing and fracture of particles captured between colliding media (beads). A challenge in the preparation of drug nanosuspensions lies in ensuring their physical stability, a critical factor for their successful integration into drug delivery systems [2,9,10,11]. While achieving stability in drug nanosuspensions is of paramount importance, this challenge has been addressed through careful and strategic selection, as well as screening of stabilizers and their concentrations [2,12,13,14,15].

WSMM, nonetheless, comes with inherent drawbacks such as being time-consuming, costly, and energy-intensive [16]. Moreover, while recognizing issues like potential product contamination due to bead wear, process-induced solid-state changes, and prolonged milling times required for drug nanosuspension preparation, the pharmaceutical nanotechnology literature has focused on the stabilization of drug nanosuspensions [17,18,19], with relatively scant information on process modeling and the resolution of the processing challenges [20]. Given that breakage kinetics govern the cycle time and production rate for achieving a desired fineness, the development and optimization of WSMM as well as resolution of the above-mentioned problems necessitate a profound understanding of breakage kinetics and its controlling process–design parameters. In this regard, the size and material of the beads affect breakage kinetics significantly: there exists an optimal bead size, and ceramic beads enable faster milling than crosslinked polystyrene beads [21,22]. Regarding the process parameters, an increase in the stirrer speed enhances the breakage rate, evident from smaller median sizes and 90% passing sizes of particles at any given milling time during WSMM [1,8,23,24]. While higher bead loading increases the breakage rate [8,23,25,26], an increase in drug loading reduces it albeit with an improvement in operational efficiency [23,27]. In contrast to other process parameters, suspension flow rate and batch size of a recirculating WSMM have been rarely examined in terms of their impact on the breakage kinetics and PSD evolution; hence, investigation of their potential impacts is within the scope of this paper. 

A quantitative understanding of the impacts of the process–design parameters through modeling, particularly mechanistic or first-principles-based modeling, can be highly beneficial in process development and optimization of the WSMM process [20,28]. Various mechanistic models, including computational fluid dynamics (CFDs) [29], discrete element model (DEM) [30,31], population balance model (PBM) [32,33,34], microhydrodynamic (MHD) model [35,36], coupled methods (CFD–DEM, CFD–PBM, etc.) [37,38,39,40], and the stress intensity–stress number model [41,42] have been employed for modeling the WSMM process. 

Population balance models (PBMs) are often used for simulation, control, and optimization of a wide variety of particulate processes [43,44]. In addition to their capability to describe the spatial and/or temporal evolution of the particle size distribution (PSD), they have also been utilized to identify particle breakage mechanisms [45,46]. While several PBM studies exist for the simulation of batch WSMM operation [34] and multi-pass continuous WSMM operation [47], Annapragada and Adjei [32] offered the first credible PBM structure for simulating a recirculating WSMM in the literature, wherein dextrose particles were milled to yield an aerosol suspension (not a nanosuspension). Their model assumed perfect mixing in both the milling chamber and the holding tank. Unfortunately, they did not measure or model the evolution of the entire PSD to validate their model. In addition, contrary to the authors’ assertions, the model prediction deviated significantly from the limited experimental data. Their simulations of a recirculating WSMM, without any experimental verification, suggested that faster breakage occurred when a higher rotor speed, higher suspension flow rate, and smaller beads were used, whereas slightly finer PSDs were obtained when bead loading was increased in a narrow range (80–85% *v*/*v*). Some of these findings are in line with previous WSMM studies (refer to the review [2]); however, the significant positive impact of the suspension flow rate is quite surprising, which has not been verified experimentally. Also, they did not investigate the impact of the batch size. Both aspects will be scrutinized in the current study with experimental verification.

In this study, we aim to elucidate potential impacts of batch size, suspension volumetric flow rate, and imperfect mixing in a recirculating stirred mill. A cell-based PBM, which reduces to the PBM in [32] for one cell (perfect mixing), was developed. First, four different specific breakage rate functions were fitted to experimental PSD data at baseline conditions assuming a well-mixed mill. Then, the PSD evolution at various batch sizes and flow rates was simulated using the best function to compare with respective experimental data and validate the PBM. Furthermore, potential impact of imperfect mixing in the mill chamber was simulated by considering more than one cell and different back-mixing flow ratios of the cell-based PBM. The analysis of the simulation results along with experimental verification will allow us to develop a fundamental understanding of recirculating WSMM operation, providing valuable guidance for process development.

## 2. Experimental

### 2.1. Materials

The drug utilized in this study was fenofibrate (FNB), which was obtained in BP grade from Jai Radhe Sales (Ahmedabad, India). Fenofibrate is considered a poorly water-soluble drug, with an aqueous solubility of 0.8 mg/L at room temperature [24]. Hydroxypropyl cellulose (HPC) of L grade, donated by Nisso America Inc. (New York, NY, USA), was used as a non-ionic polymeric stabilizer. An anionic surfactant, sodium dodecyl sulfate (SDS) of ACS grade, was purchased from GFS chemicals (Columbus, OH, USA). Zirmil Y grade yttrium-stabilized zirconia (YSZ) beads from Saint Gobain ZirPro (Mountainside, NJ, USA) with a density of 6000 kg/m^3^ and a nominal size of 400 μm were used as grinding media. The actual median size of the beads (405 μm) was measured using a laser diffraction technique in dry dispersion mode, employing a Helos/Rodos particle size analyzer (Sympatec, Pennington, NJ, USA).

### 2.2. Preparation and Characterization Methods

**Figure 1:**
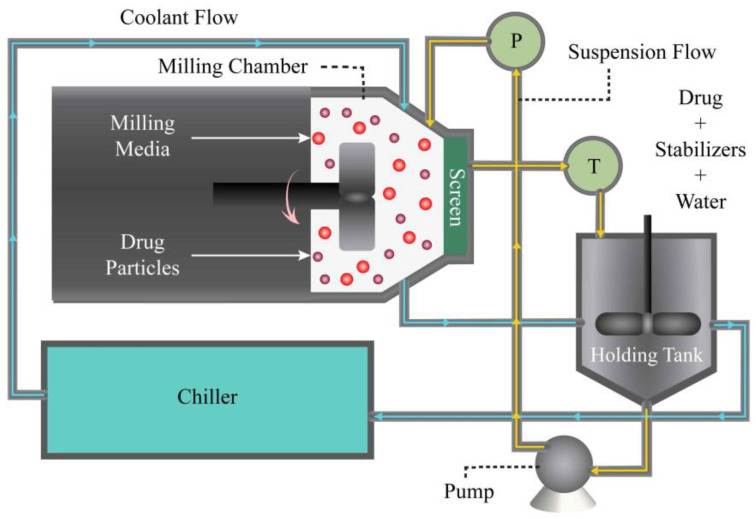
A schematic of the recirculation mode of wet stirred media milling (WSMM).

To prepare an FNB pre-suspension, hydroxypropylcellulose-L (HPC-L) and sodium dodecyl sulfate (SDS) were dissolved in 200 mL of deionized water first, followed by dispersion of the FNB powder using a shear mixer (Cat.# 14-503, Fisher Scientific, Pittsburgh, PA, USA). The mixer ran at a speed of 300 rpm for 2 h. The formulation composition of the suspension was determined in the view of previous research [35]: 10% FNB, 7.5% HPC-L, and 0.05% SDS. After preparation, the pre-suspension was stored at a temperature of 8 °C overnight. Subsequently, the pre-suspension was then transferred to a Microcer mill (Netzsch Fine Particle Size Technology, LLC, Exton, PA, USA) and ground for a duration of *t* = 180 min at a stirrer speed of 3000 rpm and bead loading of *c* = 0.5 (*v*/*v*). Bead loading *c* was determined by taking the ratio of the true volume of the beads to the volume of the milling chamber (*V*_m_ = 80 mL) on a volumetric basis (*v*/*v*). The actual volume occupied by the beads was used to determine the bead loading. To ensure continuous recirculation of the suspension (Figure 1), a peristaltic pump (Cole-Palmer, Master Flex, Vermont Hills, IL, USA) was employed. The pump facilitated the flow of the suspension between the holding tank and the milling chamber at a volumetric flow rate of *Q* = 126 mL/min for the baseline conditions (Table 1). To retain the beads within the milling chamber, a stainless-steel screen with openings half the size of the nominal bead size (200 μm) was utilized. This screen served as a barrier to prevent the beads from exiting the milling chamber while allowing for the suspension to flow through. To remove heat generated during the milling, a chiller (Model M1-.25A-11HFX, Advantage Engineering, Greenwood, IN, USA) was employed. The chiller effectively cooled the setup, ensuring that the temperature remained under control throughout the milling process. In 4 additional experiments, batch sizes and suspension flow rates were doubled and halved from their respective baseline values to examine their impacts (see Table 1).

The volume-based PSD of the drug suspensions at different milling times was determined using laser diffraction with an LS 13–320 Beckman Coulter instrument (Brea, CA, USA). Samples were collected from the outlet of the mill at specific time intervals, denoted as 2^s^, where s represents the time interval (s = 0, 1, 2, …, 7), along with additional samples taken at 24, 48, 96, and 180 min. The final sample was obtained from the holding tank. Prior to each measurement, approximately 1.0 mL of the suspension sample was diluted with 5.0 mL of the stabilizer solution using a vortex mixer (Fisher Scientific Digital Vortex Mixer, Model No: 945415, Pittsburgh, PA, USA) at 1500 rpm for one minute. During the measurements, polarized intensity differential scattering (PIDS) was maintained between 40% and 50%, while the obscuration remained below 8%. The PSD was determined by the instrument’s software, which utilized the Mie scattering theory, considering refractive indices of 1.55 for FNB and 1.33 for water. The measurements were repeated four times, and the average PSD was used in the model fits. 

## 3. Theoretical

### 3.1. Preliminaries: Residence Time Distribution

In continuous WSMM, the product PSD is affected by the residence time distribution (RTD) of the particles in the mill depending also on the specific mode of operation: single pass, multi-pass, and recirculation (circuit) [48,49]. To measure RTD function experimentally, a tracer pulse is introduced at the mill’s inlet point, and the tracer’s concentration is then recorded at the outlet of mill in single-pass operation mode [50,51,52]. Space time, which equals the average residence time if there are no dead zones and bypass in the milling chamber, can be calculated using *τ*_s_ = *V*_m_(1 − *c*)/*Q*. For a single-pass continuous mill, an increase in *τ*_s_ results in a finer PSD; a plug-flow regime yields a narrower and somewhat finer PSD in the mill than the perfect-mixing regime [53,54]. It should be noted that the average residence time in the mill for a recirculation operation *τ*_c_ can be regarded as the effective milling time and is described by *τ*_c_ = *N*_to_*τ_s_*, where *N*_to_ is the number of turnovers defined by *N*_to_ = *Qt*/*V*_s_. 

The RTD of particles yields information about the transport behavior and back-mixing extent, which affect the product PSD [49]. Previous RTD studies on single-pass WSMM demonstrated that (i) the RTD function is close to that of perfect mixing especially for short mills with small length-to-diameter *L/D* ratio [33] and (ii) longer mills exhibit deviations from perfect mixing (imperfect mixing), which can be explained by either the convection–dispersion model with the Peclet number or the cell-based RTD model with (internal) recirculation between the cells [33]. The Peclet number for a single-pass WSMM is defined by Pe_s_ = *u*_a_*L/*$\overset{´}{D}$, where *u_a_*, *L*, and $\overset{´}{D}$ stand for the axial interstitial velocity of the drug suspension, length of the mill chamber, and dispersion coefficient; *u_a_* relates to the axial superficial velocity *u*_s_ through *u*_a_ = *u*_s_/(1 − *c*), where *u*_s_ = *Q/A*_c_ and *A*_c_ denote the cross-sectional area of the mill chamber perpendicular to the suspension flow. Two limiting cases reflect ideal mixing regimes: Pe_s_ = 0 (perfect mixing) and Pe_s_ → ∞ (plug flow, no axial back-mixing). Experimental RTD studies [33,49] suggest 0.6 < Pe_s_ < 5, which signifies that perfect mixing is a decent approximation in a single-pass WSMM, albeit with some deviations for long mills.

### 3.2. Formulation of the PBM

Initially introduced by Whiten [55], the cell-based RTD model has been used as an alternative to the convection–dispersion model [33,47,49,56,57,58,59]. The mill is conceptualized as an assembly of *n* well-mixed cells with recirculation between them (internal recirculation) superposed on the recirculating bulk flow of the suspension. In particular, the cell-based RTD model explicitly incorporates an axial recirculation rate of the suspension $\dot{R}$ between adjacent cells in the mill chamber. A dimensionless axial back-mixing ratio *R* is defined as *R* = $\dot{R}$/Q, which modulates the extent of back-mixing or imperfect mixing, along with *n*. Kwade and Schwedes [49] conducted a significant study affirming the accuracy of the cell-based RTD model in fitting the RTD of a WSMM. In their investigation, the number of cells equaled the number of stirrer discs, and a single parameter—the back-mixing ratio *R*—was fitted to the measured RTD. Notably, this estimation of *R* through the cell-based model did not include the evolution of the PSD because milling was not considered. Further advancements in the use of the cell-based PBM approach were presented by Fadhel et al. [57] and Frances [33]. These researchers employed a modified cell-based PBM to fit experimental steady-state PSDs in WSMM; however, a recirculating WSMM with transient operation has not been modeled.

We adapted the cell-based PBM developed by [54,55] and tailored it to model a recirculating WSMM. The final cell-based PBM for suspension in the mill chamber and the holding tank can be recorded as

(1) $$\frac{dM_{i,z}}{dt}=-S_{i}M_{i,z}+\sum_{j=1}^{i-1}b_{ij}S_{j}M_{j,z}+\left\{\begin{array}{c} \frac{M_{i,T}-M_{i,z}+R\left(M_{i,z+1}-M_{i,z}\right)}{\tau_{cell}}\;if\;z=1\\ \frac{M_{i,z-1}-M_{i,z}+R\left(M_{i,z-1}+M_{i,z+1}-{2M}_{i,z}\right)}{\tau_{cell}\;}\;if\;1<z<n\\ \frac{\left(1+R\right)\left(M_{i,z-1}-M_{i,z}\right)}{\tau_{cell}\;}\;if\;z=n \end{array}\right.$$

(2) $$\frac{dM_{i,T}}{dt}=\frac{\left(M_{i,n}-M_{i,T}\right)}{\tau_{T}}$$

where *z* and T refer, respectively, to the cell index that varies from 1 to *n* and the holding tank; *i* and *j* are the size-class indices. Equations (1) and (2) pose a set of *N* × *n* ordinary differential equations (ODEs) with the following initial conditions: *M_i,z_*(0) = *M_i,_*_T_(0) = *M_i_*_,ini_ at time *t* = 0. Size classes 1 and *N* contain the coarsest particles and finest particles, respectively. *M_i_*, *S_i_*, and *b_ij_* stand for the mass concentration of (drug) particles in size class *i*, the specific breakage rate function, and the breakage distribution function, respectively. *S_i_* describes the rate at which particles in size class *i* are broken, whereas *b_ij_* represents the mass fraction of particles broken from size class *j* into size class *i*. The latter function is related to its cumulative counterpart *B_ij_* through *b_ij_* = *B_ij_* − *B_i_*_+1*j*_. Both functions depend on particle size *x_i_*. The parameter $\tau_{cell}$, denoting residence time within the cell, is defined as $\tau_{cell}=\tau_{s}/n.$ Similarly, $\tau_{T}$, representing the average residence time within the holding tank, is defined as $\tau_{T}{=V}_{T}/Q$, where *V*_T_ is the holding tank volume. The first term on the right-hand-side of Equation (1) explains the rate of disappearance of particles in size class *i* due to breakage. The second term explains the rate of appearance of progeny particles in size class *i* due to breakage of all large particles in size classes *j* < *i* (*x_j_* > *x_i_*). The last term in curly brace accounts for particle transport rates between any generic cell *z* and adjacent cells *z* − 1 and *z* + 1 due to recirculating bulk flow quantified by *Q* and internal recirculation flow quantified by $\dot{R}$. *R* is the dimensionless axial back-mixing ratio. Equation (1) accounts for finite axial mixing/dispersion in a long mill for a corresponding single-pass operation via *R* and *n*, while Equation (2) simulates the dynamics in the holding tank. The axial particle mixing/dispersion arises from the random motion of particles–beads along the longitudinal axis of a mill with an effective length *L* and/or radial velocity variations. The model allows for the investigation of limiting cases, such as zero axial back-mixing (plug flow) and perfect mixing, by considering scenarios with a large number of cells or a single well-mixed cell, respectively. The model structure is akin to that in ref. [32] when perfect mixing is assumed for short mills (z = *n* = 1).

### 3.3. Functional Forms of S_i_ and B_ij_

The specific breakage rate function *S_i_* and the cumulative breakage distribution function *B_ij_* play an important role in fitting and simulating the breakage of particles in WSMM using the PBM. The following normalized power–law function was used to describe *B_ij_*:

(3) $$B_{ij}={\left(\frac{x_{i-1}}{x_{j}}\right)}^{a_{1}}$$

where the exponent $a_{1}$ sets the slope of the *B_ij_* function in a log–log plot against the normalized particle size. Such a simple function was successfully used in the PBM of WSMM before [60]; it allows us to minimize the number of parameters to fit. It is well-established in the milling literature that the *B_ij_* function is relatively insensitive to the process parameters as compared with the *S_i_* function, which is a strong function of the process parameters [60,61]. The following *S_i_* functions, referred to as Models A–D, with different mathematical complexity and representation of different breakage kinetics were used:

(4) $$\mathrm{Model}\;A:\;S_{i}=A{\left(\frac{x_{i}}{x_{0}}\right)}^{m}$$

(5) $$\mathrm{Model}\;B:\;\left\{\begin{array}{c} S_{i}=A{\left(\frac{x_{i}}{x_{0}}\right)}^{m}\;\mathrm{for}\;x_{i}>x^{*}\\ S_{i}=0\;\mathrm{for}\;x_{i}\leq x^{*} \end{array}\right.$$

(6) $$\mathrm{Model}\;C:\;S_{i}=A{\left(\frac{x_{i}}{x_{0}}\right)}^{m}\frac{1}{1+exp[1-s_{f}\log\left(\frac{x_{i}}{x^{*}}\right)]}$$

(7) $$\mathrm{Model}\;D:\;S_{i}=A{\left(\frac{x_{i}}{x_{0}}\right)}^{m}\frac{1}{1+exp[-s_{f}(x_{i}-x^{*})]}$$

In Equations (4)–(7), *A* is the specific breakage rate constant of particles with $x_{0}$ size, *m* is the breakage rate exponent, $x_{0}$ is the normalizing reference particle size and was set to particle size of class 1 *x*_1_. In Equations (6) and (7), *s*_f_ is defined as the shape factor of the respective logistic function. Note that the power–law model, Model A, is one of the most used models in the milling literature and has been successfully used for a multitude of materials in various types of mills including the WSMM [60,62,63]. However, other WSMM investigations [8,64,65,66] experimentally identified the existence of a grinding limiting size, which is typically reported in terms of the cumulant size of the PSD; below this size, no particle size reduction takes place. However, to the best knowledge of the authors, the notion or phenomenon of a grinding limit or limiting size has not been incorporated into an *S_i_* function explicitly before. Bilgili et al. [34] observed a transition of the *S_i_* during the WSMM of a magenta pigment; however, such transition was not modeled using any of the above *S_i_* functions. Hence, in this study, commensurate with the notion of a grinding limit, we hypothesize that there exists a transition particle size x* below which the *S_i_* decreases to 0 sharply (Model B) or gradually yet much more drastically than what a typical power–law model predicts through the logistic functions (Models C and D). Models C and D are identical except for the way the difference between *x_i_* and x* was expressed: logarithmic vs. linear. 

### 3.4. A Full Back-Calculation Method for the Estimation of PBM Parameters

The parameters of the *S_i_* and *B_ij_* functions were assumed invariant in different runs (Table 1) because all process parameters, except for the suspension flow rate and the batch size, were kept constant. We hypothesize that while the suspension flow rate and the batch size can affect the PSD evolution, they do not affect the breakage functions unlike stirrer speed, bead loading, etc. Therefore, we first attempted to find the most appropriate *S_i_* model among Models A–D by fitting the PBM at the baseline experimental conditions (Run 1), as will be detailed in the next section. Since our Microcer mill is a small lab-scale stirred mill with *L/D* < 1, its RTD is expected to follow perfect mixing closely [33], and thus we set *n* = 1. Then, using the calibrated PBM, we predicted the impacts of batch size and volumetric flow rate on the PSD evolution (Runs 2–5) and compared them to the experimental PSD evolution, which allowed us to validate the PBM. Note that we did not examine the impacts of the stirrer speed, bead loading/size, and bead type in this study partly because they were experimentally studied previously [21,23,27] and partly their consideration would entail a more elaborate experimental study and a PBM structure with more than 8 parameters, which is beyond the scope of this paper.

To estimate the breakage parameters of the PBM and discriminate Models A–D, a full back-calculation method was adopted here [67]. This method employs a dynamic global optimizer–ODE solver to fit the PBM to the experimental PSD data, and therefore estimate the parameters. Specifically, the GlobalSearch function and fmincon solver within the MATLAB 2022a software [68] were utilized to minimize the following sum-of-squared residuals *SSR* between the experimentally determined PSDs in Run 1 and the model-predicted PSDs:

(8) $$SSR=\sum_{p=1}^{P}\sum_{i=1}^{K}{\left(\bar{m_{iz,p}^{MOD}}-\bar{m_{iz,p}^{EXP}}\right)}^{2}$$

where $\bar{m_{iz,p}^{MOD}}$ and $\bar{m_{iz,p}^{EXP}}$ denote, respectively, the simulated and the experimental mass fraction density distributions; *p* is the time index, *P* is the total number of PSD sampling (time points), and *K* = 94 is the number of size classes in the laser diffraction measurements. In the fitting, we specifically used *z* = *n* = 1 (perfect mixing) and fitted the PSDs at all time points (*P* = 10), except for 1 min and 2 min due to the onset of initial aggregation, as will be discussed in Section 4. The MATLAB function “GlobalSearch” was used to generate the next set of initial guesses for the next trial point using the scatter search method [69]. Throughout this paper, the PSD represented by the mass fraction density distribution in any cell *z*, i.e., $\bar{m_{iz},}$ was calculated from the mass fraction *m_iz_* as per Equations (9) and (10). Assuming the particle density remains invariant with particle size, the experimental volume-based PSD was taken as the mass-based PSD in Equation (8). Readers are referred to Appendix A for the numerical details of optimization–parameter estimation.

(9) $$m_{iz}=M_{iz}/\sum_{i=1}^{N}M_{iz}$$

(10) $$\bar{m_{iz}}=m_{iz}/\log\;(x_{i}/x_{i+1})$$

### 3.5. Impacts of the Batch Size, Flow Rate, and Imperfect Mixing and PBM Validation

After fitting the PBM to the PSD evolution of Run 1 (baseline conditions), determining its parameters, and discriminating Models A–D, we used the best *S*_i_ model in various simulations to examine the impacts of batch size (Runs 2 and 3), volumetric flow rate (Runs 4 and 5), and the degree of imperfect mixing as modulated by *R* and *n* (Runs 6–17). The detailed design of the simulation study is outlined in Table 2, providing a structured overview of the parameters and conditions examined in the simulations. As the evolution of PSD in Runs 2–5 was also studied experimentally, these runs allow us to test the predictive capability of the model and validate it. 

Note that Runs 6–17 have identical process–design parameters as those of Run 1, except that they correspond to different theoretical single-pass RTD in the milling chamber. They enable a theoretical investigation of the impacts of imperfect mixing or deviation from perfect mixing in the milling chamber because *n* = 1 in Runs 1–5 signifies perfect mixing in a theoretical single pass of a continuous mill. In the absence of recirculation from a holding tank, for a single pass of the suspension through a continuous mill, *n* = 1 corresponds to perfect mixing. An increase in *n* or a decrease in *R* for a single-pass continuous mill represents a decrease in axial back-mixing, which implies an approach to plug flow in the milling chamber [54]. For *R* = 0 (traditional *n*-equal volume tank in series RTD), even when *n* was increased from 1 to 5, the product PSD was closer to that for plug flow than that for perfect mixing, and for *n* = 15 and *n* = 60, plug-flow behavior was approached and reproduced, respectively [54]. All these findings are for a theoretical single-pass continuous mill without recirculation from the holding tank; therefore, the simulations in Table 2 will shed light on the impact of (single-pass) imperfect mixing on the evolution of the PSD in a recirculating WSMM. 

## 4. Results and Discussion

### 4.1. Estimating the Breakage Parameters and Discrimination of Models A–D

**Figure 2:**
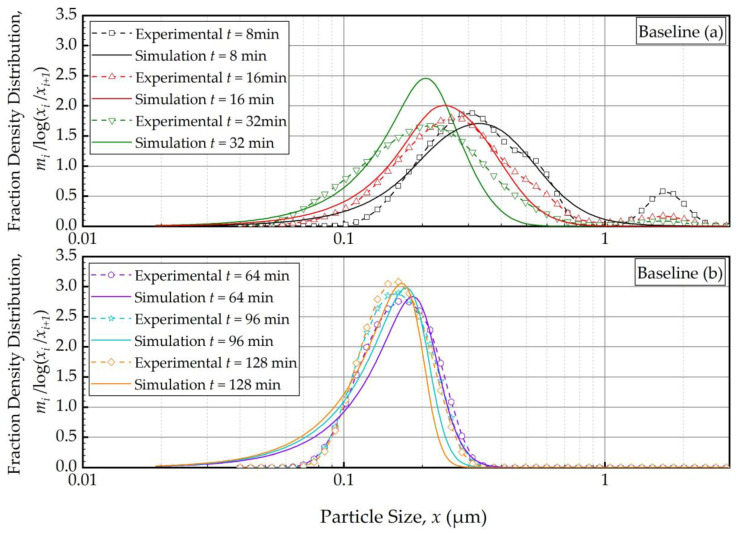
Temporal variation of the mass fraction density distribution during (**a**) the first 32 min of milling and (**b**) thereafter for the baseline experiment: volumetric flow rate of 126 mL/min and batch volume of 236 mL (Run 1). Simulation: PBM with Model C and its fitted parameters.

We fitted the PBM to the PSD evolution of Run 1 (baseline conditions) using Models A–D for *S_i_*, estimated the breakage parameters, and discriminated Models A–D based on their *SSR.* As will be elaborated in the sequel, Model C turned out to be the best *S_i_* model with the lowest *SSR*. Figure 2a,b shows the PBM simulation with Model C (with fitted parameters for *N*_T_ = 1000) and experimental PSD evolution over the initial 32 min and 64–128 min, respectively. Only selected time points are shown for clarity, e.g., the feed PSD (*t* = 0 min) is excluded for proper scaling of Figure 2. The PSD evolution starting with the feed PSD is illustrated in Figure S1 (Supplementary Materials) for the sake of completeness. Unless otherwise indicated, the PSDs refer to the suspensions at the mill outlet.

In general, the experimental PSD shifted from right to left, toward finer particles, monotonically as milling was continued (Figure 2). The modal (peak) size also shifted to the left. The feed had the 10%, 50%, and 90% passing sizes of the cumulative PSD of *x*_10_ = 5.64 ± 0.04 μm, *x*_50_ = 12.3 ± 0.05 μm, and *x*_90_ = 22.2 ± 0.06 μm. Upon 128 min of milling, these characteristic particle sizes were drastically reduced to *x*_10_ = 0.103 ± 0.001 μm, *x*_50_ = 0.149 ± 0.001 μm, and *x*_90_ = 0.212 ± 0.000 μm, suggesting a size reduction ratio of ~80. The PBM reasonably predicted these changes, albeit with some notable deviations from the experimental observations, especially at earlier milling times. For example, despite the unimodal PSD of the feed suspension, a bimodal PSD was observed, with a minor mode in the 1–3 μm range, during the first 16 min, which completely disappeared with prolonged milling (after 32 min). Figure S1 illustrates both the unimodal feed PSD and evolving bimodality during the first 8 min. Nevertheless, it is worth noting that the disparity between the simulation and experimental results gradually diminished after the 16-min mark. This is why 1 min and 2 min PSDs were disregarded from the model fitting. This bimodality could be explained by the presence of a mixture of aggregates and primary drug particles due to the early onset of aggregation. The simulation failed to accurately capture the observed bimodality in the experimental data because the PBM did not account for aggregates that formed during the first 16 min (see Figure 2 and Figure S1). Note that as fine particles were formed, they might have aggregated as polymer–surfactant adsorption is a kinetic process that takes time. This mechanism was supported by the fact that the bimodality disappeared after 32 min as sufficient time was given for adsorption. Moreover, the simulation aligned more closely with the experimental data, indicating an improvement in its fitting capability as the milling progressed. Between 64 min and 128 min (Figure 2b), the PSD became narrower, and the extent of size reduction decreased as the grinding limit was approached. The characteristic sizes at 180 min were *x*_10_ = 0.102 ± 0.008 μm, *x*_50_ = 0.145 ± 0.007 μm, and *x*_90_ = 0.210 ± 0.014 μm, which were almost identical to those at 128 min. This fact further supports our hypothesis that with increasing milling time, the stabilizer adsorption progressed while mitigating aggregation, and an apparent grinding limit was attained. In fact, in our earlier study, 1-week stability testing with the wet-milled FNB suspensions demonstrated that the same HPC–SDS combination provided effective stabilization during the storage [35]. Readers are referred to [35] for details of this extensive physical stability study. Overall, Model C fitted the PSD data reasonably well especially for sufficiently long milling times or in the sub-micron region (Figure 2b). The slight deviations in the sub-100 nm region could originate from modeling error, but it may also be due to the limitations of the laser diffraction method for accurately measuring sub-100 nm sizes despite the equipment’s use of the PIDS technology.

The breakage parameters, estimated by the GlobalSearch algorithm using Model C, are presented in Table 3. The table includes results for varying numbers of trials, i.e., sets of guessed parameters (*N*_T_), alongside the lower and upper boundaries, as well as the initial guesses of parameters. Interestingly, an increase in *N*_T_ from 200 up to 1000 did not cause any change in the estimated parameters and *SSR*. Hence, we conclude that a probable global optimum was reached even at low *N*_T_. On the other hand, *SSR* was reduced, and the parameters were altered when *N*_T_ was increased for Models A, B, and D (refer to Tables S1–S3 of Supplementary Materials). For all models, a probable globally optimal solution was reached at *N*_T_ = 1000 (MATLAB’s default *N*_T_) as both the *SSR* and the estimated parameters remained invariant when *N*_T_ was increased from low values to 1000. Specifically, the *SSR* and the estimated parameters did not change after 200 (Model C), 400 (Model D), and 800 (Models A and B) trials. Regardless, the parameter estimates were taken from *N*_T_ = 1000 for all models because a higher *N*_T_ correlates with a higher probability of having identified a global minimum [70]. 

The outcomes of the parameter estimation for the PBM with Models A–D are presented in Table 4, which allow for direct model discrimination. PSD evolution plots are also presented for Models A, B, and D in Figures S6–S8 of Supplementary Materials for their comparison with Model C (Figure 2). Notably, Model C was the best model with the lowest *SSR*. Models C and D had similar *SSR*, which was significantly lower than those of Models A and B. This suggests a similarity in the performance of Models C and D, as can also be seen from a comparison of Figure 2 and Figure S8, making them practically interchangeable. This is not surprising because Models C and D have a similar mathematical structure: a product of power–law and logistic functions. Conversely, Model A, the traditional power–law model, had 41% higher *SSR* than Model C. Although Model B significantly improved upon Model A, the fitted PSD evolution with Model B exhibited a discontinuity in the PSD profile that was absent from the experimental data (Figure S7). Hence, besides their lower *SSR*, Models C and D were preferred over Model B because of the absence of any discontinuity in their fitted PSD profiles (see Figure 2 and Figure S8). In contrast to most *S_i_* parameters of the PBM, the exponent *a*_1_ of *B_ij_* function was insensitive to *S_i_* model structure: *a*_1_ = 2.33 ± 0.09 (3.9% RSD). In particular, the PBMs with different *S_i_* models predict similar breakage distribution/mechanisms of particles. While this is intuitively expected, the optimization-based back calculation is known to be notoriously plagued with interactions between *S_i_* and *B_ij_* functions in parameter estimation, which can lead to inaccurate estimations when a local optimization scheme is used [59]. The small variation of the *a*_1_, and thus *B_ij_*, for different *S_i_* models used in the PBM could be attributed to the use of a global optimization scheme, giving further credence to the methods used. 

**Figure 3:**
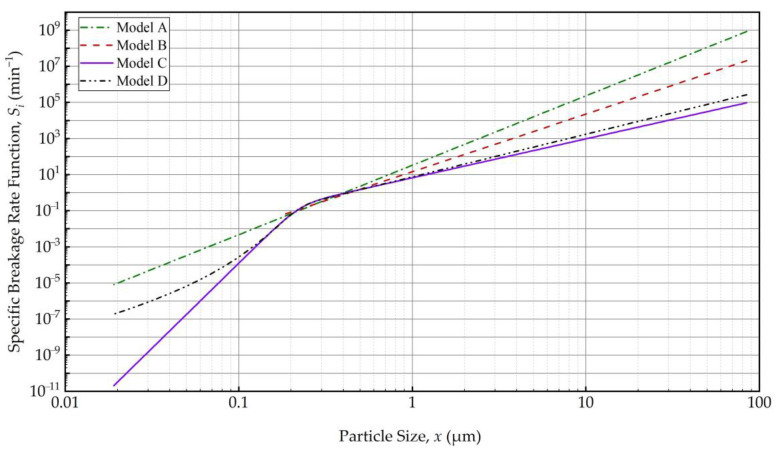
The variation of the specific breakage rate *S_i_* with the particle size *x_i_* according to the fitted Models A–D. *S_i_* equals 0 for *x_i_* ≤ x* per Model B, which is not shown on this log–log plot.

Models B–D indicated a transition particle size x* of 195 ± 20 nm (10.2% RSD), which commensurate with the notion of a grinding limit size. This finding is in line with [35], where a rather simple *n*th-order kinetic model of the WSMM of fenofibrate suspensions under similar milling conditions indicated a grinding limiting size of 142 nm. This interesting phenomenon, first time explored via PBM simulations here, is illustrated in Figure 3. Model A (power–law) exhibited a linear variation with slope *m* on the log–log plot, whereas the models with x* predicted lower *S_i_* and a drastic slope change at x*. The decrease in *S_i_* from about 200 nm to 20 nm was remarkable for Models C and D as compared to that for Model A. As compared with the transition for Model B (step function), the transition was smooth for Models C and D, which explains the absence of discontinuity in the PSD evolution (refer to Figure 2 and Figure S8 vs. Figure S7). However, Model C shows a sharper drop in *S_i_* with a decrease in particle size below x*, which is more aligned with the existence of a grinding limit. The fitting results overall suggest the following: (i) a PBM without consideration of a transition particle size x* will not be able to describe the breakage phenomenon in nanomilling of drugs accurately and (ii) a novel function, which is a product of power–law and logistic functions, signifying the existence of a transition particle size commensurate with a grinding limit, fitted the evolution the best. 

PBM simulations also suggest that the PSD in the holding tank was coarser than the mill outlet PSD within the first 16 min and these two PSDs became the same thereafter until the end (Figure S9). This finding implies that after a certain milling time, whether the suspension sample for PSD analysis is taken from the tank or the outlet is immaterial. 

### 4.2. Prediction of the Impacts of Batch Size and Flow Rate Using Model C

#### 4.2.1. Impact of Batch Size and Flow Rate

**Figure 4:**
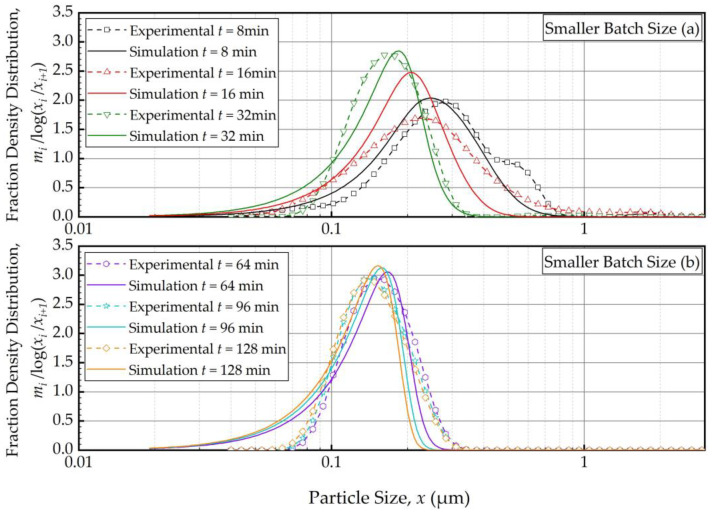
Temporal variation of the mass fraction density distribution during (**a**) the first 32 min of milling and (**b**) thereafter for the smaller batch size experiment: volumetric flow rate of 126 mL/min and batch volume of 118 mL (Run 2).

**Figure 5:**
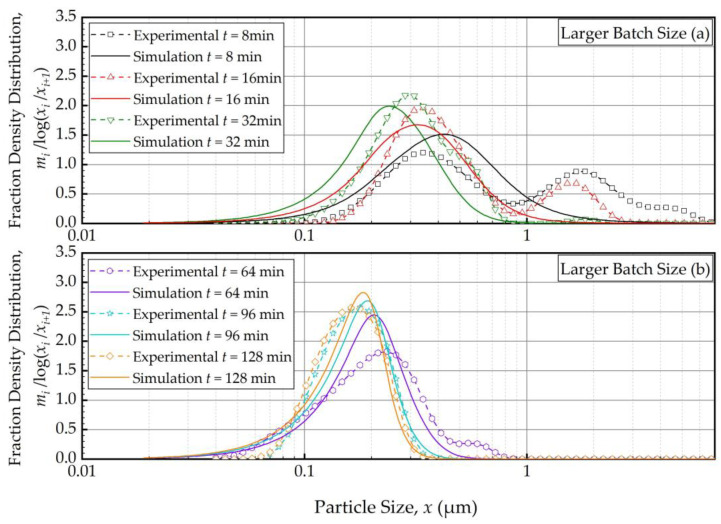
Temporal variation of the mass fraction density distribution during (**a**) the first 32 min of milling and (**b**) thereafter for the larger batch size experiment: volumetric flow rate of 126 mL/min and batch volume of 472 mL (Run 3).

As the PBM with Model C fitted the baseline conditions (Run 1) best, we used this model and its estimated parameters in Table 4 to simulate the impacts of the batch size and the suspension flow rates (Runs 2–5). Figure 4 and Figure 5 present the PBM-predicted and experimental PSD evolution when the batch size was halved (Run 2) and doubled (Run 3) from the baseline value of 236 mL, respectively, keeping the suspension flow rate at 126 mL/min. The predictions of the PBM in Runs 2 and 3 had *SSR* values of 71.3 and 79.1, respectively, surpassing the *SSR* of the fitting of Run 1 data, i.e., 38.0. This was also evident from a visual comparison of the deviations of the PBM fitting/prediction and the experimental data in Figure 2, Figure 4 and Figure 5. Albeit being undesirable, this increase in *SSR* was expected as the PBM parameters were kept the same in the predictions (Runs 2 and 3) as in the fitting (Run 1); they were not re-fitted to experimental data in Runs 2 and 3 wherein the batch size was drastically altered. Despite the elevated *SSR*, Figure 4 and Figure 5 illustrate the following: (i) an increase in batch size led to notably slower evolution of the PSD, i.e., slower milling of the whole batch and (ii) the PBM predicted the general evolution trend reasonably well, except for the bimodality. The initial bimodality became more evident and sustained up to 64 min when the batch size was increased. As usual, the bimodality disappeared upon prolonged milling in the sub-micron range.

**Figure 6:**
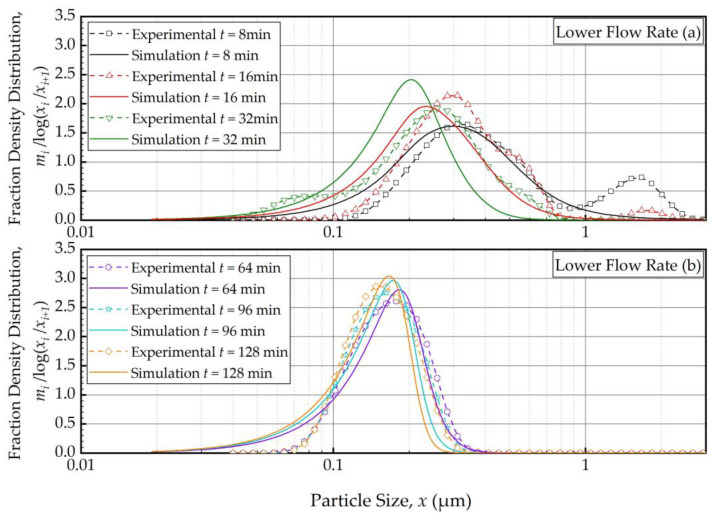
Temporal variation of the mass fraction density distribution during (**a**) the first 32 min of milling and (**b**) thereafter for the lower flow rate experiment: volumetric flow rate of 63 mL/min and batch volume of 236 mL (Run 4).

**Figure 7:**
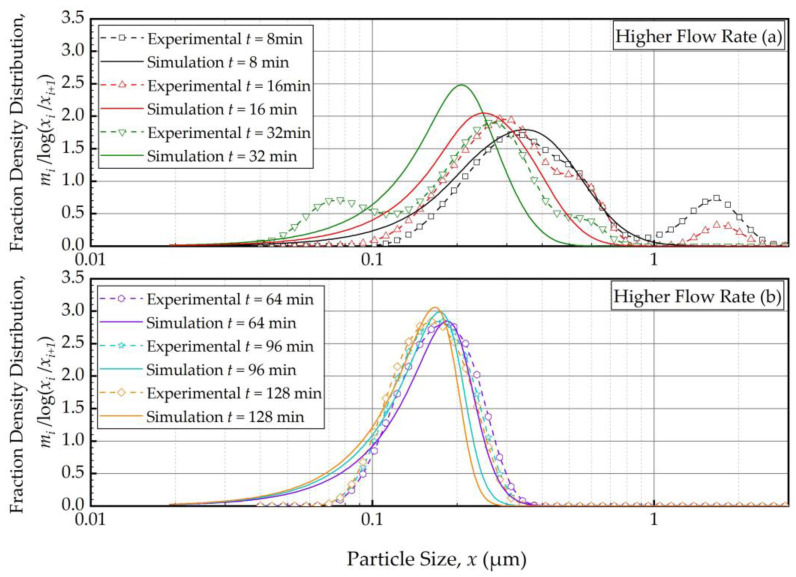
Temporal variation of the mass fraction density distribution during (**a**) the first 32 min of milling and (**b**) thereafter for the higher flow rate experiment: volumetric flow rate of 250 mL/min and batch volume of 236 mL (Run 5).

Figure 6 and Figure 7 present the PBM-predicted and experimental PSD evolution when the suspension flow rate was halved (Run 4) and doubled (Run 5) from the baseline value of 126 mL/min, respectively, keeping the batch size at 236 mL. The predictions of the PBM in Runs 4 and 5 had *SSR* values of 40.9 and 40.8, respectively, which is slightly above the *SSR* of the fitting of Run 1 data, i.e., 38.0. The suspension flow rate had an insignificant effect on the PSD evolution, as can be seen from a visual comparison in Figure 2, Figure 6 and Figure 7.

**Figure 8:**
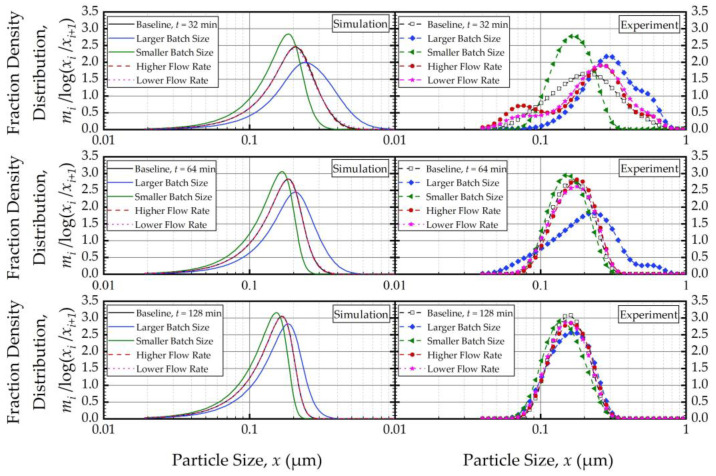
Experimentally measured and simulated impacts of the batch size and volumetric flow rate on the mass fraction density distribution at 32, 64, and 128 min.

To elucidate the impacts of the batch size and the suspension flow rate, the predictions and experimental observations were presented for only 32, 64, and 128 min (Figure 8). In general, one weakness of the current PBM was its inability to explain the bi-modal PSD, especially prevalent during the earlier part of the milling. However, it could capture several salient features of the recirculating WSMM process and the impacts of the process parameters. For any given set of process conditions, as intuitively expected, both the PBM simulations and the experimental data showed a finer PSD for longer milling; hence, the desirable product PSD can be tailored by setting the milling time. The batch size had a dramatic impact on the PSD; in general, a smaller batch was associated with smaller particle sizes and a narrower distribution, indicating a more homogeneous product. Only after a prolonged milling (128 min), the PSD of the larger batch approached those of the baseline and the smaller batches. In contrast, the effect of volumetric flow rate on the PSD was found to be rather small, and mainly notable at 32 min at which bi-modality associated with particle aggregation had a confounding effect. The effect of the volumetric flow rate was not examined experimentally in [32]; however, their simulations indicated a significant impact. In our study, both the simulations and the experiments showed a relatively insignificant impact. The differences may stem from the differences in mill design, materials’ formulations, and preparation of micron-sized suspensions vs. nanosuspensions. We will not further speculate why the simulations in [32] showed a notable impact of the volumetric flow rate unlike our simulations; however, it suffices to mention that our experiments support the conclusion based on the simulations. The origin of this insignificant impact of the volumetric flow rate becomes clear when the effective milling time is examined in the next section. 

#### 4.2.2. Milling Time vs. Effective Milling Time

**Figure 9:**
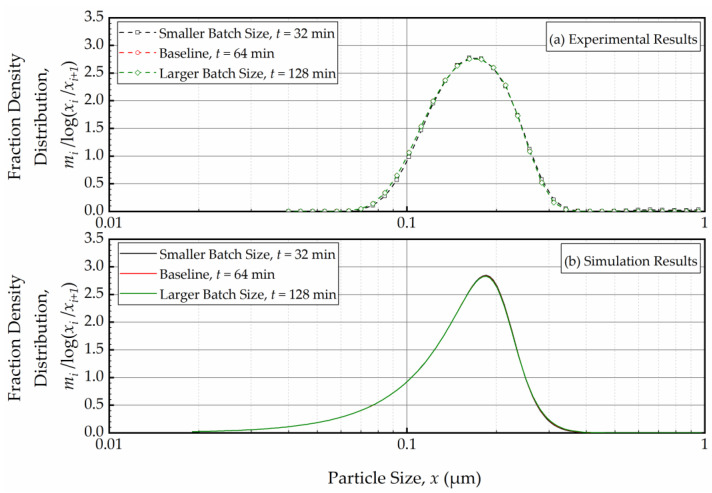
(**a**) The experimentally measured and (**b**) simulated mass fraction density distribution for various batch sizes (*V*s = 118 mL, *V*s = 236 mL, and *V*s = 472 mL in Runs 2, 1, and 3, respectively) at the same effective milling time of 10.8 min.

In the context of a recirculating WSMM, the effective milling time is different from the actual milling time; it equals the average residence time in the mill during circuit operation *τ*_c_ and can be expressed by *τ*_c_ = *N*_to_*τ_s_* = *V*_m_(1 − *c*)*t*/*V_s_*. Hence, an increase in batch size *V*_s_ causes a lower *τ*_c_ due to lower number of turnovers, which in turn leads to slower breakage of the whole batch overall. This explains the slower milling operation for the larger batches as illustrated in Figure 8. Note that *N*_to_ is directly proportional to the volumetric flow rate, whereas *τ_s_* is inversely proportional to it. Hence, *τ*_c_ becomes independent of the volumetric flowrate, which explains the insignificant impact of the volumetric flow rate on the PSD evolution (refer to Figure 8). A practical implication of these findings is that *τ*_c_ must be kept the same so that the PSD remains invariant when the batch size is changed, or the process is scaled-up. Let us verify this principle via simulations and experiments by comparing the PSD at the same effective milling time *τ*_c_. To this end, for the same *V*_m_ and *c*, the milling time *t* was selected in such a way to ensure identical *t/V*_s_ when the batch size *V*_s_ was varied. Figure 9 depicts the experimental (a) and simulation (b) results for the PSDs in the smaller batch (118 mL), the baseline (236 mL), and the larger batch (512 mL) conditions at the same effective milling time of 10.8 min, which corresponds to the actual milling times of 32 min, 64 min, and 128 min. At the same effective milling time, the PSDs at various batch sizes were identical, which was confirmed by both the experiments and the PBM simulations. Although the PBM simulations did not perfectly match the experimental PSDs, as discussed before, the PSDs were similar, and the PBM predicted the same outcome for different batch sizes, which gives further credence to the PBM.

**Figure 10:**
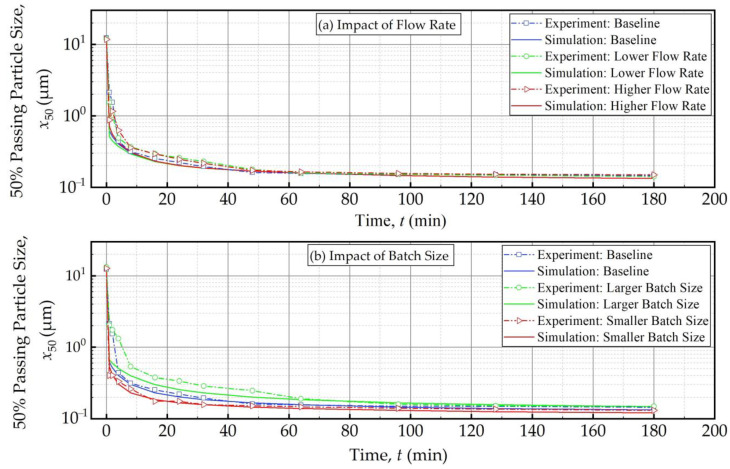
The measured and simulated evolution of the 50% passing (median) size *x*_50_ as a function of milling time: (**a**) influence of the flow rate and (**b**) influence of the batch size (Runs 1–5).

**Figure 11:**
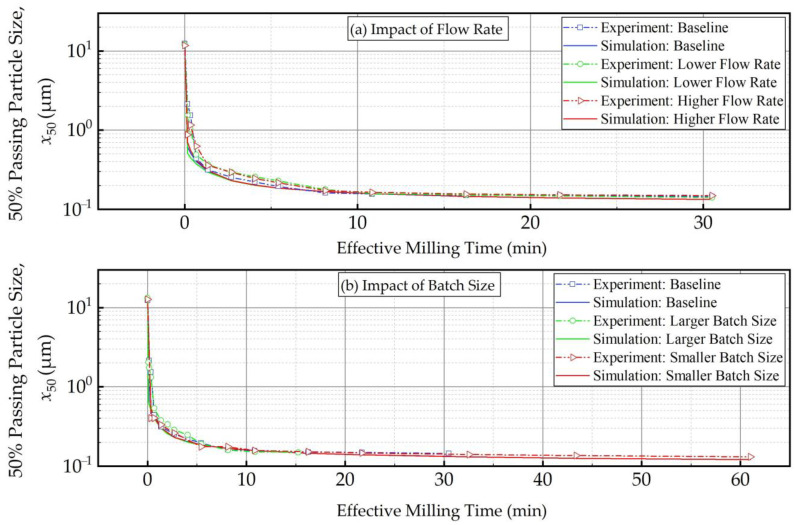
The measured and simulated evolution of the 50% passing (median) size as a function of effective milling time: (**a**) influence of the flow rate and (**b**) influence of the batch size (Runs 1–5).

Figure 10 and Figure 11 present the experimental data and simulation results for the evolution of the 50% passing (median) size *x*_50_ as a function of the milling time and the effective milling time, respectively. As expected, *x*_50_ decreased drastically during the first 32 min, and the size reduction slowed later on with an approach to the grinding limiting size (Figure 10). The insignificant impact of the suspension flow rate and the faster decrease in *x*_50_ for smaller batch were evident. What is most impressive is that when the *x*_50_ evolution is expressed in terms of the effective milling time, the curves corresponding to different batch sizes coincided within experimental accuracy (Figure 11). The impact of the suspension flow rate was relatively insignificant regardless of whether *x*_50_ evolution was expressed in terms of the milling time or the effective milling time. Overall, we conclude that the PSD equivalency established for 10.8 min effective milling time (Figure 9) is generally applicable during the whole milling, thus further supporting the use of effective milling time in process scale-up and/or batch size changes.

### 4.3. Simulating the Impact of Imperfect Mixing in the Milling Chamber

Up to this point, the PBM assumed perfect mixing in the milling chamber for a theoretical single pass, which is the expected RTD response for a small mill with a low *L/D* ratio [33]. However, for larger mills with high *L*/*D* ratio (>1), the RTD behavior for a single-pass continuous operation deviates from perfect mixing [33,49]. The cell-based PBM accounts for this deviation via *n* > 1 and fine-tunes the RTD behavior via the back-mixing ratio *R*. Interestingly, for a recirculating WSMM, the impact of *n* and *R* (different RTD behavior) on the PSD evolution has not been examined in the milling literature. Hence, in this study, we investigated the influence of *n* at two different *R* using simulations: *R* = 0 (Runs 6–9, no internal recirculation between the cells in the milling chamber) and *R* = 2.52 (Runs 10–13, strong internal recirculation between cells in the milling chamber). 

**Figure 12:**
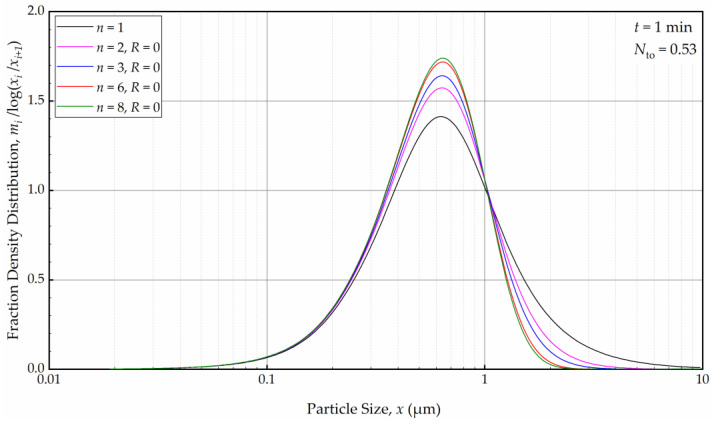
The effects of number of cells *n* at the back-mixing ratio *R* of 0 on the mass fraction density distribution at *t* = 1 min.

As depicted in Figure 12, in the absence of recirculation between the cells (*R* = 0), an increase in the number of cells *n* resulted in a narrower and sharper PSD initially (at *t* = 1 min, within the first turnover). Here, the prediction by the perfect-mixing model (*n* = 1) was included as a reference. This finding is similar to what was observed in a single-pass continuous mill without a holding tank [54]. 

**Figure 13:**
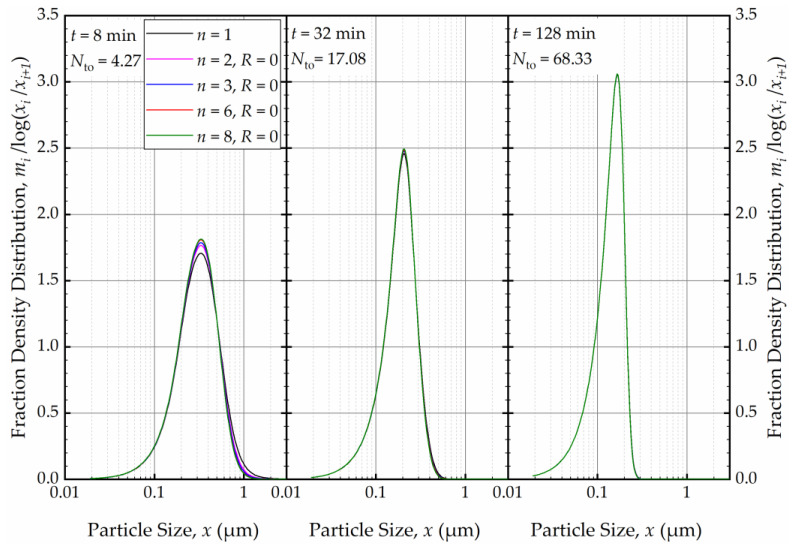
The effects of number of cells *n* at the back-mixing ratio *R* of 0 on the mass fraction density distribution at *t* = 8 min, *t* = 32 min, and *t* = 128 min.

As the milling was prolonged, as illustrated in Figure 13, the impact of *n* disappeared as early as 8 min, and at the end of 32 min of milling, all PSDs converged to the same PSD. The bulk flow emanating from the holding tank dominates the RTD of the circuit rapidly and any imperfect-mixing effect within the milling chamber was nullified upon an increase in the number of turnovers *N*_to_. Although there is no internal recirculation between the cells (*R* = 0), the recirculating bulk flow in the circuit from/to holding tank dominates the mixing behavior and the RTD of the circuit [49]. Hence, any RTD difference for a single-pass operation of the same mill, as modulated by *n* and *R* in the milling chamber, has a diminishing effect on the PSD upon an increase in milling time and number of turnovers. One practical implication of this theoretical finding for pharmaceutical engineers is that performing rigorous, elaborate RTD studies for the continuous stirred mills, typically carried out in single-pass mode at the steady-state, may not be warranted if the same mills are eventually used in the recirculation (circuit) mode. One can use the perfect-mixing model (*n* = 1) for the recirculating WSMM even though the single-pass operation may exhibit significant deviation of the RTD from that of the perfect mixing. Any deviations for such modeling appear to be restricted to early milling times; however, this finding is expected to depend on other system parameters especially *V*_s_ and *V*_m_, which were 236 mL and 80 mL in these simulations (Runs 6–17). 

**Figure 14:**
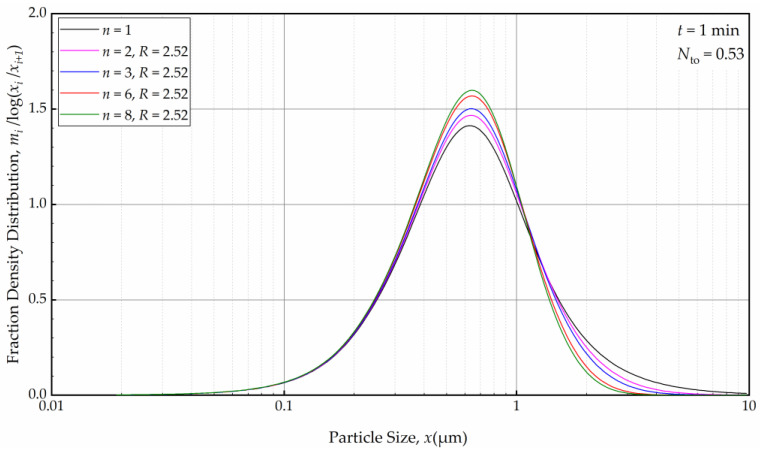
The effects of number of cells *n* at the back-mixing ratio *R* of 2.52 on the mass fraction density distribution at *t* = 1 min.

**Figure 15:**
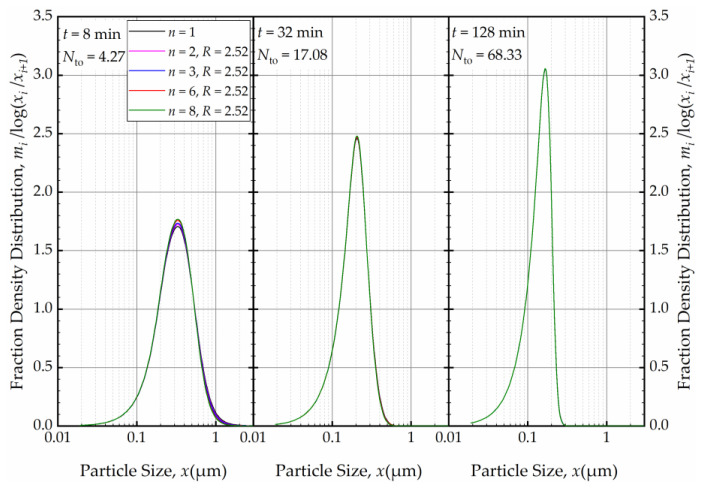
The effects of number of cells *n* at the back-mixing ratio *R* of 2.52 on the mass fraction density distribution at *t* = 8 min, *t* = 32 min, and *t* = 128 min.

Subsequently, we explored the impact of *n* when the axial back-mixing ratio *R* was set to 2.52 (taken from [49] for a single-pass continuous WSMM). Figure 14 illustrates that, akin to the condition of *R* = 0, a higher *n* initially led to a narrower and sharper PSD at *t* = 1 min. A cursory look at Figure 12 and Figure 14 suggests that the impact of *n* was slightly more discernible for *R* = 0 than for *R* = 2.52. Nevertheless, Figure 15 reveals that as milling progressed, the *n* effect was nullified, and identical PSDs were obtained for different *n* at 32 min. While the PSDs at 1 min were notably different for *R* = 0 and *R* = 2.52, a comparison of Figure 13 and Figure 15 reveals that the PSDs were similar or identical for other milling times.

**Figure 16:**
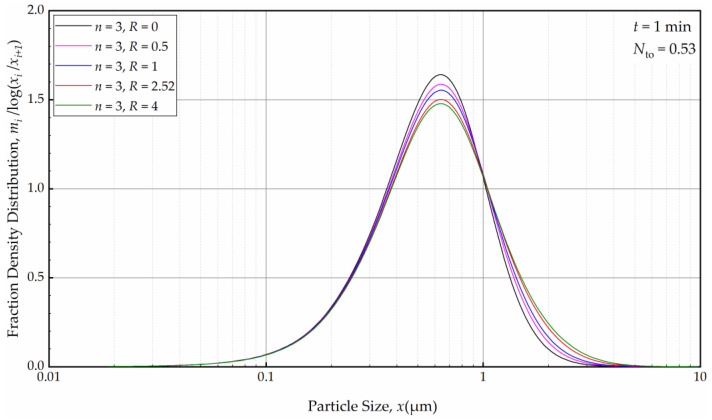
The effects of back-mixing ratio *R* for three cells on the PSD at *t* = 1 min.

**Figure 17:**
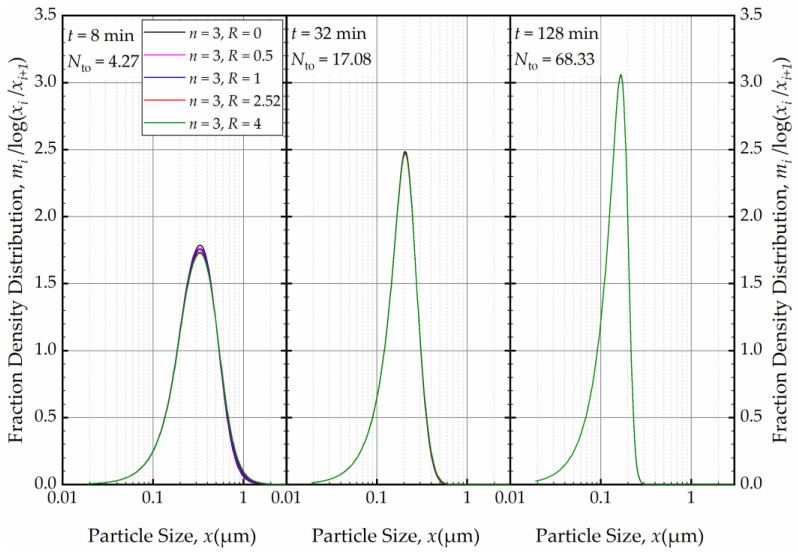
The effects of back-mixing ratio *R* for three cells on the PSD at *t* = 8 min, *t* = 32 min, and *t* = 128 min.

For a fixed *n*, e.g., *n* = 3, the effects of the axial back-mixing ratio *R* were simulated via Runs 7 and 14–17 (refer to Table 2). An increase in *R*, which indicates a greater extent of axial back-mixing emanating from the higher rate of internal recirculation between the cells, led to coarser and wider PSD at *t =* 1 min (Figure 16). This effect disappeared, similar to the effect of *n*, upon further milling and concomitantly an increase in the number of turnovers (Figure 17). The PSDs at 32, 64, and 128 min at various *n* and *R* (different RTD response of the mill for a theoretical single-pass operation) were either similar or identical. Again, this finding points out that regardless of the differences of the RTD response of the continuous mill, which is usually obtained via single-pass operation at the steady-state, the recirculating bulk flow of the suspension dominated the circuit RTD, and the impacts of *n* and *R* were insignificant after a few turnovers. In particular, the back-mixing in the mill becomes insignificant in a recirculating WSMM operation after a few passes of the suspension through the mill.

Although we did not simulate *n* > 8, we expect that the invariant PSDs observed after 32 min will not change even when higher *n* values are used. As established in ref. [54], for a single-pass continuous mill, the cell-based PBM with *n* = 60 produces a PSD that is identical to the PSD obtained for a plug-flow RTD. Our findings from the current PBM simulations imply that the PSDs in the recirculating WSMM (circuit operation) are invariant to *n* and *R* (after a few turnovers), and that they correspond to an equivalent single-pass continuous mill with plug-flow behavior approximately. This finding is in line with the sharpening of the circuit RTD upon an increase in the number of turnovers, and the approach of the circuit RTD to that of the plug flow for an equivalent single-pass mill [49]. 

### 4.4. On the Grinding Limit and the Transition Particle Size 

There are two major reasons as to why the specific breakage rate decreases significantly as particles become smaller (refer to Figure 3): (i) capturing efficiency of sub-micron particles between the relatively large beads is very low, and it becomes lower as the particles get smaller, as predicted by the microhydrodynamic model (see, e.g., [35]) and (ii) the number of defects, flaws, and pre-cracks in particles decreases with particle size, which causes a lower breakage probability for the smaller particles [71]. When a brittle–ductile transition occurs, the grinding limit appears because below that size the relaxation mechanism changes from brittle fracture to ductile (plastic) flow [64,72,73]. Our estimate of the grinding limit, typically reported as a cumulant size, for drugs is in the range of 30–90 nm, based on previous studies [8,74]. In the current study, a transition particle size x* of 195 ± 20 nm (10.2% RSD) was estimated. It is critical to note that x* of the PBM is not equal to the grinding limiting size obtained by milling for extremely long milling times (4–16 h) and/or with intensified process conditions [8,64]. Also, it is worth noting that Model C predicts a specific breakage rate range of ~10^–9^–7 × 10^–5^ min^–1^ for the size range of 30–90 nm particles (refer to Figure 3). It is speculated that with such extremely low specific breakage rates (close to 0), 30–90 nm FNB particles will not break extensively within the time scale of the milling experiments explored here, implying that the grinding limit of FNB may indeed fall within this range. To put this into perspective, 30–90 nm particles break ~10^10^–10^5^ times slower than 1 μm particles (Figure 3). We will not further explore this point as finding the true grinding limit was not the primary objective of this study.

### 4.5. Limitations of the Current PBM

The majority of the PBM’s deviation from the experimental PSD data during the early milling times originated from particle aggregation. The PBM did not consider particle aggregation as it would significantly increase the modeling complexity and the number of parameters. We did not consider the volume of the suspension in the tubing between the holding tank and the milling chamber, and the lag time associated with the flow in the tubing. Moreover, the effects of rotor speed, bead loading, and bead size were not varied in this study, which would significantly affect the *A* parameter. In that case, *A* should be multiplicatively decomposed into several power law terms for these variables, and their parameters need to be estimated first. These aspects will be investigated in a forthcoming study.

## 5. Conclusions and Outlook

We studied the impacts of batch size, suspension flow rate, and imperfect mixing during the production of fenofibrate nanosuspensions in a recirculating WSMM within the context of a cell-based PBM. Four specific breakage rate functions were discriminated by fitting the PBM to the PSD evolution at the baseline process conditions. It is concluded that Model C, which consists of a product of power–law and logistic functions, is the best model. The parameter estimation study revealed the criticality of incorporating a transition particle size commensurate with the notion of a grinding limit. The fitted PBM was then used to predict the salient features of the impacts of the batch size and the suspension flow rate. Both the experiments and the predictions demonstrated that an increase in batch size entails a proportionate increase in milling time to keep the PSD invariant, and this can be carried out by keeping the same residence time in the mill for the circuit operation. This circuit residence time was regarded as the effective milling time that must be kept identical upon batch size increase and in process scale-up. The suspension flow rate was found to have insignificant impact on the PSD. An intriguing finding from the PBM simulations was that no matter what the single-pass RTD of the mill, as modulated by various *n* and *R* of the cell-based PBM, the PSD is not affected by them after a few turnovers in the recirculation mode. This finding practically implies that detailed RTD studies may not be warranted for modeling a recirculating WSMM. As aggregation was present in the system, future modeling effort is warranted to include aggregation terms in the PBM to improve the fitting–prediction capability. Also, the PBM should be expanded to account for the impacts of all process parameters. Despite its limitations, the presented PBM offered significant insights about recirculating WSMM, also providing valuable guidance for process development and scale-up. 

## Figures and Tables

**Table 1:** Conditions of the milling experiments.

Run No.	Identifier	Batch Size, *V*_s_ (mL)	Volumetric Flow Rate, *Q* (mL/min)
1	Baseline	236	126
2	Smaller Batch Size	118	126
3	Larger Batch Size	472	126
4	Lower Flow Rate	236	63
5	Higher Flow Rate	236	250

**Table 2:** Various simulation parameters used in the cell-based PBM.

Run No.	Simulated Effect	Batch Size, *V*_s_ (mL)	Volumetric Flow Rate, *Q* (mL/min)	No. of Cells, *n* (-)	Back-Mixing Ratio, *R* (-)
1	Baseline	236	126	1	–
2	Smaller Batch Size	118	126	1	–
3	Larger Batch Size	472	126	1	–
4	Lower Flow Rate	236	63	1	–
5	Higher Flow Rate	236	250	1	–
6	No. of Cells *n*, *R* = 0	236	126	2	0
7		236	126	3	0
8		236	126	6	0
9		236	126	8	0
10	No. of Cells *n*, *R* = 2.52	236	126	2	2.52
11		236	126	3	2.52
12		236	126	6	2.52
13		236	126	8	2.52
14	Back-mixing Ratio *R*, *n* = 3	236	126	3	0.5
15		236	126	3	1
16		236	126	3	2.52
17		236	126	3	4

**Table 3:** Fitted parameters of Model C, lower and upper boundaries of the parameters, initial guess, and sum-of-squared residuals *SSR* for different numbers of trials *N*_T_.

	Model C	$a_{1}$	$A{(min}^{-1})$	m	$x^{*}(\mu m)$	*s_f_*
	Lower Boundary	0	50	0	0.038	5
	Upper Boundary	5	10^8^	4	1	30
	Initial Guess	2	5000	2	0.1	20
*N*_T_ = 200	Fitted Parameters	2.31	2.72 × 10^6^	2.15	0.196	16.7
	*SSR*	38.0				
*N*_T_ = 400	Fitted Parameters	2.31	2.72 × 10^6^	2.15	0.196	16.7
	*SSR*	38.0				
*N*_T_ = 800	Fitted Parameters	2.31	2.72 × 10^6^	2.15	0.196	16.7
	*SSR*	38.0				
*N*_T_ = 1000	Fitted Parameters	2.31	2.72 × 10^6^	2.15	0.196	16.7
	*SSR*	38.0				

**Table 4:** The estimated breakage parameters for different specific breakage functions and associated *SSR* values (*N*_T_ = 1000).

**Model A**	$a_{1}$	$A{(min}^{-1})$	m		
Fitted Parameters	2.46	3.32 × 10^11^	3.84		
*SSR*	53.5				
**Model B**	$a_{1}$	$A{(min}^{-1})$	m	$x^{*}(\mu m)$	
Fitted Parameters	2.25	2.92 × 10^9^	3.19	0.174	
*SSR*	43.4				
**Model C**	$a_{1}$	$A{(min}^{-1})$	m	$x^{*}(\mu m)$	***s*** **_f_**
Fitted Parameters	2.31	2.72 × 10^6^	2.15	0.196	16.7
*SSR*	38.0				
**Model D**	$a_{1}$	$A{(min}^{-1})$	m	$x^{*}(\mu m)$	$s_{f}\;{(\mu m}^{-1})$
Fitted Parameters	2.31	1.03 × 10^7^	2.36	0.214	41.7
*SSR*	38.4				

## Data Availability

The data are contained within the article and its Supplementary Materials.

## Supplementary Materials

The following supporting information can be downloaded at: https://www.mdpi.com/article/10.3390/pharmaceutics16030353/s1, Section S1: All supporting data about parameter estimation–optimization and evolution of the PSD; Table S1: Fitted parameters of Model A, lower and upper boundaries of the parameters, initial guess, and sum-of-squared residuals *SSR* for different numbers of trials *N*_T_; Table S2: Fitted parameters of Model B, lower and upper boundaries of the parameters, initial guess, and sum-of-squared residuals *SSR* for different numbers of trials *N*_T_; Table S3: Fitted parameters of Model D, lower and upper boundaries of the parameters, initial guess, sum-of-squared residuals *SSR* for different numbers of trials *N*_T_; Figure S1: Temporal variation of the mass fraction density distribution during (a) the first 8 min of milling, (b) between 16 and 48 min of milling, and (c) thereafter for the baseline experiment: volumetric flow rate of 126 mL/min and batch volume of 236 mL (Baseline: Run 1). Simulation: PBM with Model C and its fitted parameters. Note that 1 min and 2 min data were not used in the PBM parameter estimation due to pronounced aggregation and bimodality; Figure S2: Temporal variation of the mass fraction density distribution during (a) the first 8 min of milling, (b) between 16 and 48 min of milling, and (c) thereafter for Run 2: volumetric flow rate of 126 mL/min and batch volume of 118 mL. Simulation: PBM with Model C and its fitted parameters; Figure S3: Temporal variation of the mass fraction density distribution during (a) the first 8 min of milling, (b) between 16 and 48 min of milling, and (c) thereafter for Run 3: volumetric flow rate of 126 mL/min and batch volume of 472 mL. Simulation: PBM with Model C and its fitted parameters; Figure S4: Temporal variation of the mass fraction density distribution during (a) the first 8 min of milling, (b) between 16 and 48 min of milling, and (c) thereafter for Run 4: volumetric flow rate of 63 mL/min and batch volume of 236 mL. Simulation: PBM with Model C and its fitted parameters; Figure S5: Temporal variation of the mass fraction density distribution during (a) the first 8 min of milling, (b) between 16 and 48 min of milling, and (c) thereafter for Run 5: volumetric flow rate of 250 mL/min and batch volume of 236 mL. Simulation: PBM with Model C and its fitted parameters; Figure S6: Temporal variation of the mass fraction density distribution during (a) the first 32 min of milling and (b) thereafter for the baseline experiment: volumetric flow rate of 126 mL/min and batch volume of 236 mL (Run 1). Simulation: PBM with Model A and its fitted parameters; Figure S7: Temporal variation of the mass fraction density distribution during (a) the first 32 min of milling and (b) thereafter for the baseline experiment: volumetric flow rate of 126 mL/min and batch volume of 236 mL (Run 1). Simulation: PBM with Model B and its fitted parameters; Figure S8: Temporal variation of the mass fraction density distribution during (a) the first 32 min of milling and (b) thereafter for the baseline experiment: volumetric flow rate of 126 mL/min and batch volume of 236 mL (Run 1). Simulation: PBM with Model D and its fitted parameters; Figure S9: Simulated temporal variation of the mass fraction density distribution in the milling chamber and the holding tank during (a) the first 32 min of milling and (b) thereafter for the baseline experiment: volumetric flow rate of 126 mL/min and batch volume of 236 mL (Run 1, Model C).

## Abbreviations

Symbols used	
*A*	apparent breakage rate constant, min^–1^
*a*_1_	breakage distribution exponent
*A*_c_	cross-sectional area of the mill chamber perpendicular to suspension flow, m^2^
*b*	breakage distribution parameter
*B*	cumulative breakage distribution parameter
*c*	volume fraction of the beads in the suspension–bead mixture
CFD	computational fluid dynamics
*D*	diameter of the rotor, m
$\overset{´}{D}$	dispersion coefficient, m^2^/s
*D*_m_	mill chamber diameter, m
DEM	discrete element method
*K*	total number of size classes in the laser diffraction measurement
*L*	length of the mill, m
*m_i_*	mass fraction of particles in size class *i*
$\bar{m}$ *_i_*	mass fraction density of particles in size class *i*
*M_i_*	mass concentration in size class *i*, kg/m^3^
MHD	microhydrodynamic
*n*	number of cells in cell-based PBM
*N*	number of size classes used in the PBM
*N*_to_	number of theoretical passes or turnovers
*N*_T_	number of trials
ODE	ordinary differential equation
*P*	total number of time samples
PBM	population balance model
Pe_c_	Peclet number of a continuous mill in recirculation operation
Pe_s_	Peclet number of a single-pass continuous mill
PSD	particle size distribution
*Q*	volumetric flow rate of the recirculating suspension, m^3^/s
*R*	back-mixing ratio
$\dot{R}$	internal recirculation rate, m^3^/s
RTD	residence time distribution
*S*	specific breakage rate parameter, min^–1^
*s*_f_	shape factor
*SI*	stress intensity, Pa
*SN*	stress number
*SSR*	sum-of-squared-residuals
*t*	milling time, min
*u*_a_	axial interstitial velocity of the drug suspension, m/s
*u*_s_	superficial velocity of the drug suspension, m/s
*V*_m_	free volume of the milling chamber available for bead filling, m^3^
*V*_s_	batch volume of the suspension, m^3^
*V*_T_	volume of the suspension in the holding tank, m^3^
WSMM	wet stirred media milling
*x*	particle size, µm
*x**	transition particle size, µm
$x_{0}$	normalizing reference particle size, µm
YSZ	yttrium-stabilized zirconia
Greek Letters	
*τ*_s_	average residence time in a single pass, min
*τ*_c_	average residence time in the mill in recirculation (circuit) mode, min
*τ*_cell_	average residence time in a cell, min
*τ*_T_	average residence time in the holding tank, min
*ω*	rotational speed of the stirrer (rotor), rpm
Indices	
10, 50, 90	10%, 50%, and 90% passing sizes of the cumulative volume PSD
EXP	experimental
*i*, *j*	size-class index
m	mill chamber
MOD	model predicted
*n*	total number of cells in the cell-based PBM
*p*	time index
T	holding tank

## Appendix A

The optimization was executed for four different numbers of trial points *N_T_*: 200 as the default minimum, 400, 800, and 1000 as the MATLAB’s default to test if a probable global optimum was obtained. A Dell Precision 7820 Tower with Intel(R) Xeon(R) Gold 6226R CPU at 2.90 GHz and 2.89 GHz (two processors) was used to perform the optimization simulations. We set the termination tolerances on constraint violation, function value, and parameters to 10^–9^ in the optimizer. Relative and absolute tolerances were set to 10^–4^ and 10^–6^, respectively, in the ODE solver. In the cell-based PBM, the numbers of numerical size classes *N* and geometric progression were set to 360 and 2^1/25^, respectively, with *x*_0_ = 400 μm. The interpolation needed to interchange the PSD between the experimental size classes and the numerical size classes followed a similar approach to that in ref. [67]. The experimental feed (initial) PSD data in cumulative form with 94 size classes (*K* = 94) were interpolated to generate the PSD data for the simulation size classes (*N* = 360) using the function “pchip” in MATLAB. This cumulative PSD with 360 size classes was subsequently converted to mass fraction and mass concentration of particles in size class *i*. The mass concentration with 360 size classes was used as the initial condition at *t* = 0 for the ODE solver. After the PBM solution for the mass concentration in each simulation size class (*N* = 360) was obtained, the mass fraction was calculated using Equation (9) and then the cumulative model PSD was calculated. Using the function “pchip”, the cumulative model PSD was mapped from the simulation size classes back into the experimental size classes (*K* = 94) via interpolation to determine the cumulative and mass fraction PSDs; the latter were used to calculate mass fraction density and *SSR*.

## References

[1] Tanaka Y., Inkyo M., Yumoto R., Nagai J., Takano M., Nagata S. (2012). Nanoparticulation of Probucol, a Poorly Water-Soluble Drug, Using a Novel Wet-Milling Process to Improve in vitro Dissolution and in vivo Oral Absorption. Drug Dev. Ind. Pharm..

[2] Li M., Azad M., Davé R., Bilgili E. (2016). Nanomilling of Drugs for Bioavailability Enhancement: A Holistic Formulation-Process Perspective. Pharmaceutics.

[3] Malamatari M., Taylor K.M.G., Malamataris S., Douroumis D., Kachrimanis K. (2018). Pharmaceutical Nanocrystals: Production by Wet Milling and Applications. Drug Discov. Today.

[4] Bhakay A., Merwade M., Bilgili E., Dave R.N. (2011). Novel Aspects of Wet Milling for the Production of Microsuspensions and Nanosuspensions of Poorly Water-Soluble Drugs. Drug Dev. Ind. Pharm..

[5] Bhakay A., Rahman M., Dave R.N., Bilgili E. (2018). Bioavailability Enhancement of Poorly Water-Soluble Drugs via Nanocomposites: Formulation–Processing Aspects and Challenges. Pharmaceutics.

[6] Merisko-Liversidge E., Liversidge G.G., Cooper E.R. (2003). Nanosizing: A Formulation Approach for Poorly-Water-Soluble Compounds. Eur. J. Pharm. Sci..

[7] Peltonen L. (2018). Design Space and QbD Approach for Production of Drug Nanocrystals by Wet Media Milling Techniques. Pharmaceutics.

[8] Li M., Yaragudi N., Afolabi A., Dave R., Bilgili E. (2015). Sub-100 nm Drug Particle Suspensions Prepared via Wet Milling with Low Bead Contamination Through Novel Process Intensification. Chem. Eng. Sci..

[9] Tuomela A., Hirvonen J., Peltonen L. (2016). Stabilizing Agents for Drug Nanocrystals: Effect on Bioavailability. Pharmaceutics.

[10] Kesisoglou F., Panmai S., Wu Y. (2007). Nanosizing—Oral Formulation Development and Biopharmaceutical Evaluation. Adv. Drug Deliv. Rev..

[11] Wang Y., Zheng Y., Zhang L., Wang Q., Zhang D. (2013). Stability of Nanosuspensions in Drug Delivery. J. Control. Release.

[12] Peltonen L., Hirvonen J. (2010). Pharmaceutical Nanocrystals by Nanomilling: Critical Process Parameters, Particle Fracturing, and Stabilization Methods. J. Pharm. Pharmacol..

[13] Bitterlich A., Laabs C., Busmann E., Grandeury A., Juhnke M., Bunjes H., Kwade A. (2014). Challenges in Nanogrinding of Active Pharmaceutical Ingredients. Chem. Eng. Technol..

[14] Cerdeira A.M., Mazzotti M., Gander B. (2010). Miconazole Nanosuspensions: Influence of Formulation Variables on Particle Size Reduction and Physical Stability. Int. J. Pharm..

[15] Verma S., Huey B.D., Burgess D.J. (2009). Scanning Probe Microscopy Method for Nanosuspension Stabilizer Selection. Langmuir.

[16] Kawatra S.K. (2006). Advances in Comminution.

[17] Juhnke M., Märtin D., John E. (2012). Generation of Wear During the Production of Drug Nanosuspensions by Wet Media Milling. Eur. J. Pharm. Biopharm..

[18] Kumar S., Burgess D.J. (2014). Wet Milling Induced Physical and Chemical Instabilities of Naproxen Nano-Crystalline Suspensions. Int. J. Pharm..

[19] Sharma P., Denny W.A., Garg S. (2009). Effect of Wet Milling Process on the Solid State of Indomethacin and Simvastatin. Int. J. Pharm..

[20] Bilgili E., Guner G. (2020). Mechanistic Modeling of Wet Stirred Media Milling for Production of Drug Nanosuspensions. AAPS Pharm. Sci. Technol..

[21] Parker N., Rahman M., Bilgili E. (2020). Impact of Media Material and Process Parameters on Breakage Kinetics–Energy Consumption During Wet Media Milling of Drugs. Eur. J. Pharm. Biopharm..

[22] Li M., Alvarez P., Bilgili E. (2017). A Microhydrodynamic Rationale for Selection of Bead Size in Preparation of Drug Nanosuspensions via Wet Stirred Media Milling. Int. J. Pharm..

[23] Afolabi A., Akinlabi O., Bilgili E. (2014). Impact of Process Parameters on the Breakage Kinetics of Poorly Water-Soluble Drugs During Wet Stirred Media Milling: A Microhydrodynamic View. Eur. J. Pharm. Sci..

[24] Singh S.K., Srinivasan K., Gowthamarajan K., Singare D.S., Prakash D., Gaikwad N.B. (2011). Investigation of Preparation Parameters of Nanosuspension by Top-Down Media Milling to Improve the Dissolution of Poorly Water-Soluble Glyburide. Eur. J. Pharm. Biopharm..

[25] Patel D.J., Patel J.K., Pandya V.M. (2010). Improvement in the Dissolution of Poorly Water-Soluble Drug Using Media Milling Technique. Thai J. Pharm. Sci..

[26] Nakach M., Authelin J.-R., Agut C. (2017). New Approach and Practical Modelling of Bead Milling Process for the Manufacturing of Nanocrystalline Suspensions. J. Pharm. Sci..

[27] Ghosh I., Schenck D., Bose S., Ruegger C. (2012). Optimization of Formulation and Process Parameters for the Production of Nanosuspension by Wet Media Milling Technique: Effect of Vitamin E TPGS and Nanocrystal Particle Size on Oral Absorption. Eur. J. Pharm. Sci..

[28] Toneva P., Peukert W., Salman A.D., Ghadiri M., Hounslow M.J. (2007). Modelling of Mills and Milling Circuits. Handbook of Powder Technology.

[29] Winardi S., Widiyastuti W., Septiani E., Nurtono T. (2018). Simulation of Solid-Liquid Flows in a Stirred Bead Mill Based on Computational Fluid Dynamics (CFD). Mater. Res. Express.

[30] Gudin D., Turczyn R., Mio H., Kano J., Saito F. (2006). Simulation of the Movement of Beads by the Discrete Element Method (DEM) with Respect to the Wet Grinding Process. AIChE J..

[31] Gudin D., Kano J., Saito F. (2007). Effect of the Friction Coefficient in the Discrete Element Method Simulation on Media Motion in a Wet Bead Mill. Adv. Powder Technol..

[32] Annapragada A., Adjei A. (1996). Numerical Simulation of Milling Processes as an Aid to Process Design. Int. J. Pharm..

[33] Frances C. (2004). On Modelling of Submicronic Wet Milling Processes in Bead Mills. Powder Technol..

[34] Bilgili E., Hamey R., Scarlett B. (2006). Nano-Milling of Pigment Agglomerates Using a Wet Stirred Media Mill: Elucidation of the Kinetics and Breakage Mechanisms. Chem. Eng. Sci..

[35] Guner G., Yilmaz D., Bilgili E. (2021). Kinetic and Microhydrodynamic Modeling of Fenofibrate Nanosuspension Production in a Wet Stirred Media Mill. Pharmaceutics.

[36] Eskin D., Zhupanska O., Hamey R., Moudgil B., Scarlett B. (2005). Microhydrodynamic Analysis of Nanogrinding in Stirred Media Mills. AIChE J..

[37] Jayasundara C.T., Yang R., Guo B., Yu A., Rubenstein J. (2009). Effect of Slurry Properties on Particle Motion in IsaMills. Miner. Eng..

[38] Jayasundara C.T., Yang R., Yu A. (2012). Effect of the Size of Media on Grinding Performance in Stirred Mills. Miner. Eng..

[39] Yamada Y., Sakai M. (2013). Lagrangian–Lagrangian Simulations of Solid–Liquid Flows in a Bead Mill. Powder Technol..

[40] Beinert S., Schilde C., Gronau G., Kwade A. (2014). CFD-Discrete Element Method Simulations Combined with Compression Experiments to Characterize Stirred-Media Mills. Chem. Eng. Technol..

[41] Cerdeira A.M., Gander B., Mazzotti M. (2011). Role of Milling Parameters and Particle Stabilization on Nanogrinding of Drug Substances of Similar Mechanical Properties. Chem. Eng. Technol..

[42] Becker M., Kwade A., Schwedes J. (2001). Stress Intensity in Stirred Media Mills and Its Effect on Specific Energy Requirement. Int. J. Miner. Process..

[43] Randolph A. (2012). Theory of Particulate Processes: Analysis and Techniques of Continuous Crystallization.

[44] Ramkrishna D. (2000). Population Balances: Theory and Applications to Particulate Systems in Engineering.

[45] Verkoeijen D., Pouw G.A., Meesters G.M., Scarlett B. (2002). Population Balances for Particulate Processes—A Volume Approach. Chem. Eng. Sci..

[46] Hounslow M. (1998). The Population Balance as a Tool for Understanding Particle Rate Processes. KONA Powder Part. J..

[47] Varinot C., Berthiaux H., Dodds J. (1999). Prediction of the Product Size Distribution in Associations of Stirred Bead Mills. Powder Technol..

[48] Kwade A. (2005). Scriptum Grinding and Dispersing with Stirred Media Mills: Research and Application. Proceedings of the 21st iPAT.

[49] Kwade A., Schwedes J., Mills S.M., Salman A.D., Ghadiri M., Hounslow M.J. (2007). Wet Grinding in Stirred Media Mills. Handbook of Powder Technology.

[50] King R.P. (2001). Modeling and Simulation of Mineral Processing Systems.

[51] Abouzeid A.-Z., Mika T., Sastry K.V., Fuerstenau D. (1974). The Influence of Operating Variables on the Residence Time Distribution for Material Transport in a Continuous Rotary Drum. Powder Technol..

[52] Austin L., Luckie P., Ateya B. (1971). Residence Time Distributions in Mills. Cem. Concr. Res..

[53] Bilgili E., Scarlett B. (2005). Numerical Simulation of Open-Circuit Continuous Mills Using a Non-Linear Population Balance Framework: Incorporation of Non-First-Order Effects. Chem. Eng. Technol. Ind. Chem. Plant Equip. Process Eng. Biotechnol..

[54] Muanpaopong N., Davé R., Bilgili E. (2022). A Cell-Based PBM for Continuous Open-Circuit Dry Milling: Impact of Axial Mixing, Nonlinear Breakage, and Screen Size. Powder Technol..

[55] Whiten W. (1974). A Matrix Theory of Comminution Machines. Chem. Eng. Sci..

[56] Berthiaux H., Heitzmann D., Dodds J.A. (1996). Validation of a Model of a Stirred Bead Mill by Comparing Results Obtained in Batch and Continuous Mode Grinding. Int. J. Miner. Process..

[57] Fadhel H.B., Frances C., Mamourian A. (1999). Investigations on Ultra-Fine Grinding of Titanium Dioxide in a Stirred Media Mill. Powder Technol..

[58] Kwade A. (1999). Wet Comminution in Stirred Media Mills—Research and Its Practical Application. Powder Technol..

[59] Lira B., Kavetsky A. (1990). Applications of a New Model-Based Method of Ball Mill Simulation and Design. Miner. Eng..

[60] Tuzun M.A. (1993). A Study of Comminution in a Vertical Stirred Ball Mill. Ph.D. Thesis.

[61] Austin A.G., Luckie P.T., Klimpel R.R. (1984). Process Engineering of Size Reduction: Ball Milling.

[62] Hasan M., Palaniandy S., Hilden M., Powell M. (2018). Simulating Product Size Distribution of an Industrial Scale VertiMill^®^ Using a Time-Based Population Balance Model. Miner. Eng..

[63] Hasan M., Palaniandy S., Hilden M., Powell M. (2017). Calculating Breakage Parameters of a Batch Vertical Stirred Mill. Miner. Eng..

[64] Knieke C., Sommer M., Peukert W. (2009). Identifying the Apparent and True Grinding Limit. Powder Technol..

[65] Cho H., Waters M.A., Hogg R. (1996). Investigation of the grind limit in stirred-media milling. Int. J. Miner. Process..

[66] Knieke C., Steinborn C., Romeis S., Peukert W., Breitung-Faes S., Kwade A. (2010). Nanoparticle production with stirred-media mills: Opportunities and limits. Chem. Eng. Technol..

[67] Muanpaopong N., Davé R., Bilgili E. (2023). A Comparative Analysis of Steel and Alumina Balls in Fine Milling of Cement Clinker via PBM and DEM. Powder Technol..

[68] The MathWorks, Inc (2022). Global Optimization Toolbox User’s Guide (R2022a).

[69] Glover F. A Template for Scatter Search and Path Relinking. Proceedings of the European Conference on Artificial Evolution.

[70] Ullrich T., Fellner D.W. Statistical Analysis on Global Optimization. Proceedings of the 2014 International Conference on Mathematics and Computers in Sciences and in Industry.

[71] Vogel L., Peukert W. (2003). Breakage Behaviour of Different Materials—Construction of a Master Curve for the Breakage Probability. Powder Technol..

[72] Boldyrev V., Pavlov S., Goldberg E. (1996). Interrelation Between Fine Grinding and Mechanical Activation. Int. J. Miner. Process..

[73] Schönert K., Steier K. (1971). Die Grenze der Zerkleinerung bei kleinen Korngrößen. Chem. Ing. Tech..

[74] Maar S., Damm C., Peukert W. (2022). Wet Nanomilling of Naproxen Using a Novel Stabilization Mechanism via Zirconium Complexation. Adv. Powder Technol..

