# Supplementary material for: A Novel PBM for Nanomilling of Drugs in a Recirculating Wet Stirred Media Mill: Impacts of Batch Size, Flow Rate, and Back-Mixing
Source: Pharmaceutics. 2024 Mar 2;16(3):353. doi: 10.3390/pharmaceutics16030353 (PMC10974007; doi:10.3390/pharmaceutics16030353)
Supplement: Supplementary file 1 [file pharmaceutics-16-00353-s001.zip › pharmaceutics-2857767-supplementary.pdf]

pharmaceutics

MDPI

Section S1. All supporting data about parameter estimation–optimization and evolution of the PSD

Table S1. Fitted parameters of Model A, lower and upper boundaries of the parameters, initial guess, and sum-of-squared residuals *SSR* for different numbers of trials N_T.

	Model A	a_1	A (min^{-1})	m
	Lower Boundary	0	500	0
	Upper Boundary	5	10^{13}	4
	Initial Guess	2	5×10^{9}	2
N_T = 200	Fitted Parameters	2.62	9.99×10^{8}	3.04
	SSR	60.7		
N_T = 400	Fitted Parameters	2.45	4.46×10^{11}	3.88
	SSR	53.5		
N_T = 800	Fitted Parameters	2.46	3.32×10^{11}	3.84
	SSR	53.5		
N_T = 1000	Fitted Parameters	2.46	3.32×10^{11}	3.84
	SSR	53.5		

Table S2. Fitted parameters of Model B, lower and upper boundaries of the parameters, initial guess, and sum-of-squared residuals *SSR* for different numbers of trials N_T.

	Model B	a_1	A (min^{-1})	m	x^* (μm)
	Lower Boundary	0	500	0	0.038
	Upper Boundary	5	10^{10}	4	1
	Initial Guess	2	5×10^{4}	2	0.1
N_T = 200	Fitted Parameters	1.95	2.54×10^{8}	2.86	0.195
	SSR	46.2			
N_T = 400	Fitted Parameters	2.07	3.95×10^{9}	3.24	0.189
	SSR	43.9			
N_T = 800	Fitted Parameters	2.25	2.92×10^{9}	3.19	0.174
	SSR	43.4			
N_T = 1000	Fitted Parameters	2.25	2.92×10^{9}	3.19	0.174
	SSR	43.4			

Table S3. Fitted parameters of Model D, lower and upper boundaries of the parameters, initial guess, sum-of-squared residuals *SSR* for different numbers of trials N_T.

	Model D	a_1	A (min^{-1})	m	x^* (μm)	s_f (μm^{-1})
	Lower Boundary	0	50	0	0.038	10
	Upper Boundary	5	10^8	4	1	50
	Initial Guess	2	5×10^4	2	0.2	30
N_T = 200	Fitted Parameters	2.31	1.15×10^6	2.03	0.228	36.6
	SSR	38.7				
N_T = 400	Fitted Parameters	2.31	1.03×10^7	2.36	0.214	41.7
	SSR	38.4				
N_T = 800	Fitted Parameters	2.31	1.03×10^7	2.36	0.214	41.7
	SSR	38.4				
N_T = 1000	Fitted Parameters	2.31	1.03×10^7	2.36	0.214	41.7
	SSR	38.4				

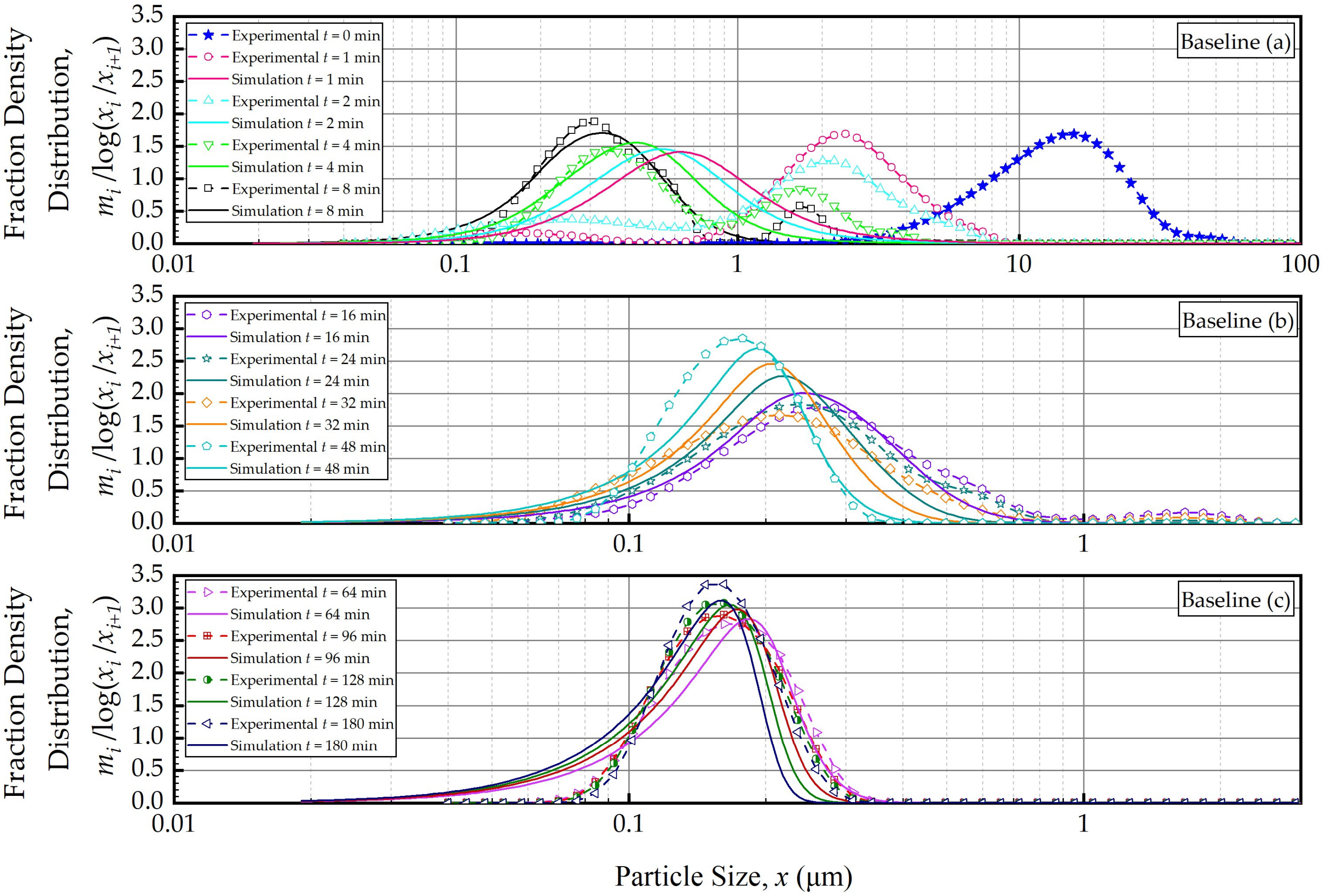

Figure S1. Temporal variation of the mass fraction density distribution during (a) the first 8 min of milling, (b) between 16 and 48 min of milling, and (c) thereafter for the baseline experiment: volumetric flow rate of 126 mL/min and batch volume of 236 mL (Baseline: Run 1). Simulation: PBM with Model C and its fitted parameters. Note that 1 min and 2 min data were not used in the PBM parameter estimation due to pronounced aggregation and bimodality.

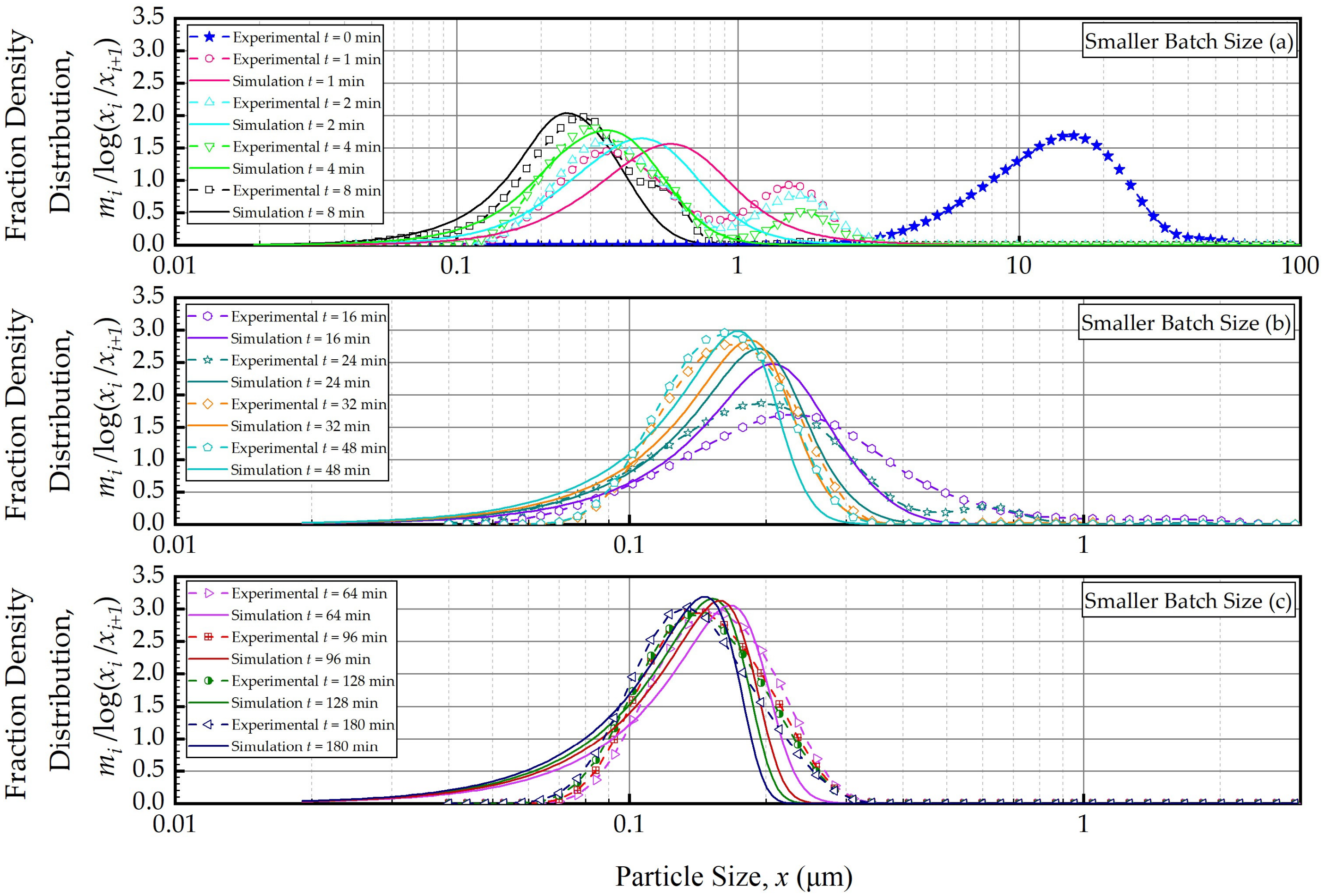

Figure S2. Temporal variation of the mass fraction density distribution during (a) the first 8 min of milling, (b) between 16 and 48 min of milling, and (c) thereafter for Run 2: volumetric flow rate of 126 mL/min and batch volume of 118 mL. Simulation: PBM with Model C and its fitted parameters.

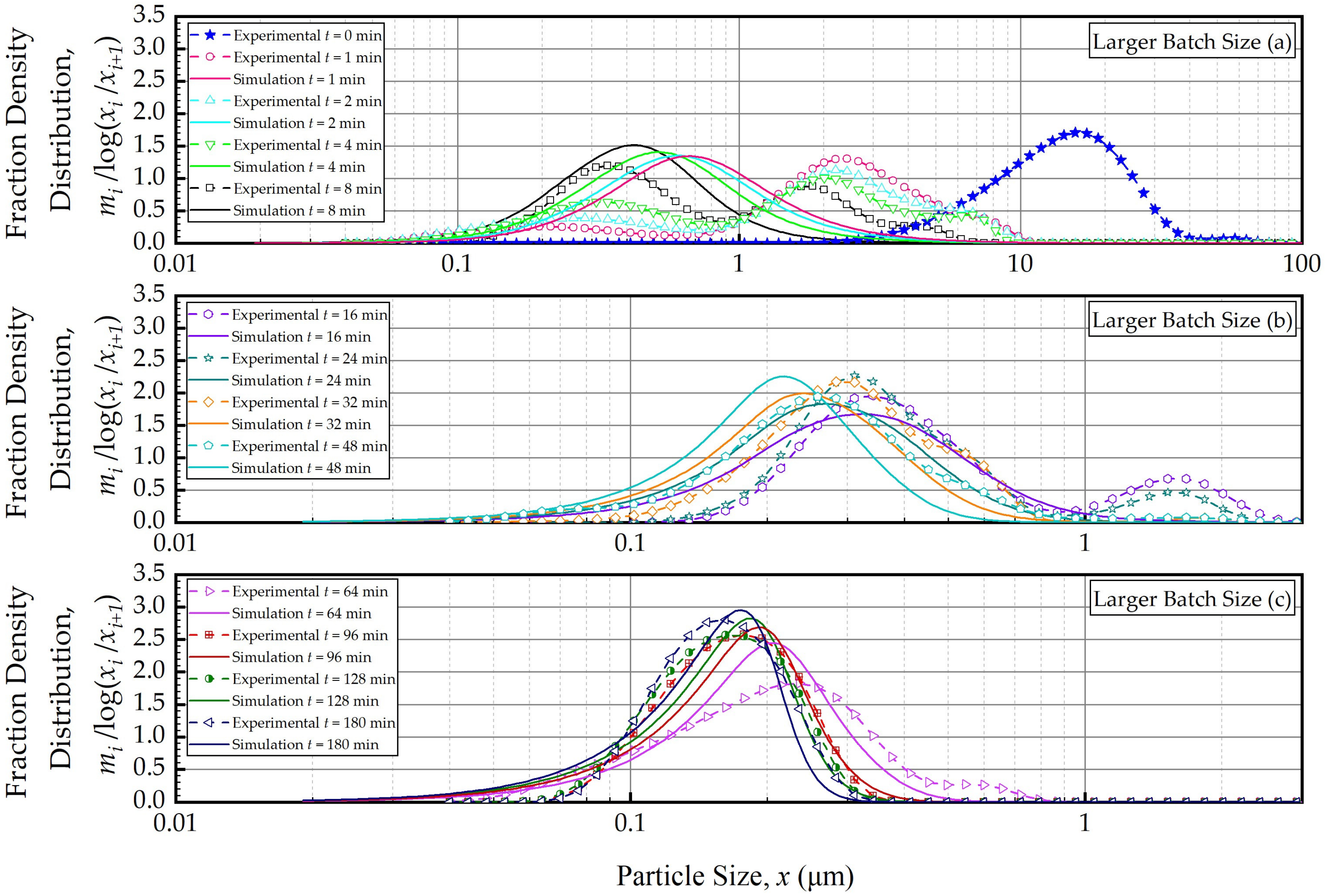

Figure S3. Temporal variation of the mass fraction density distribution during (a) the first 8 min of milling, (b) between 16 and 48 min of milling, and (c) thereafter for Run 3: volumetric flow rate of 126 mL/min and batch volume of 472 mL. Simulation: PBM with Model C and its fitted parameters.

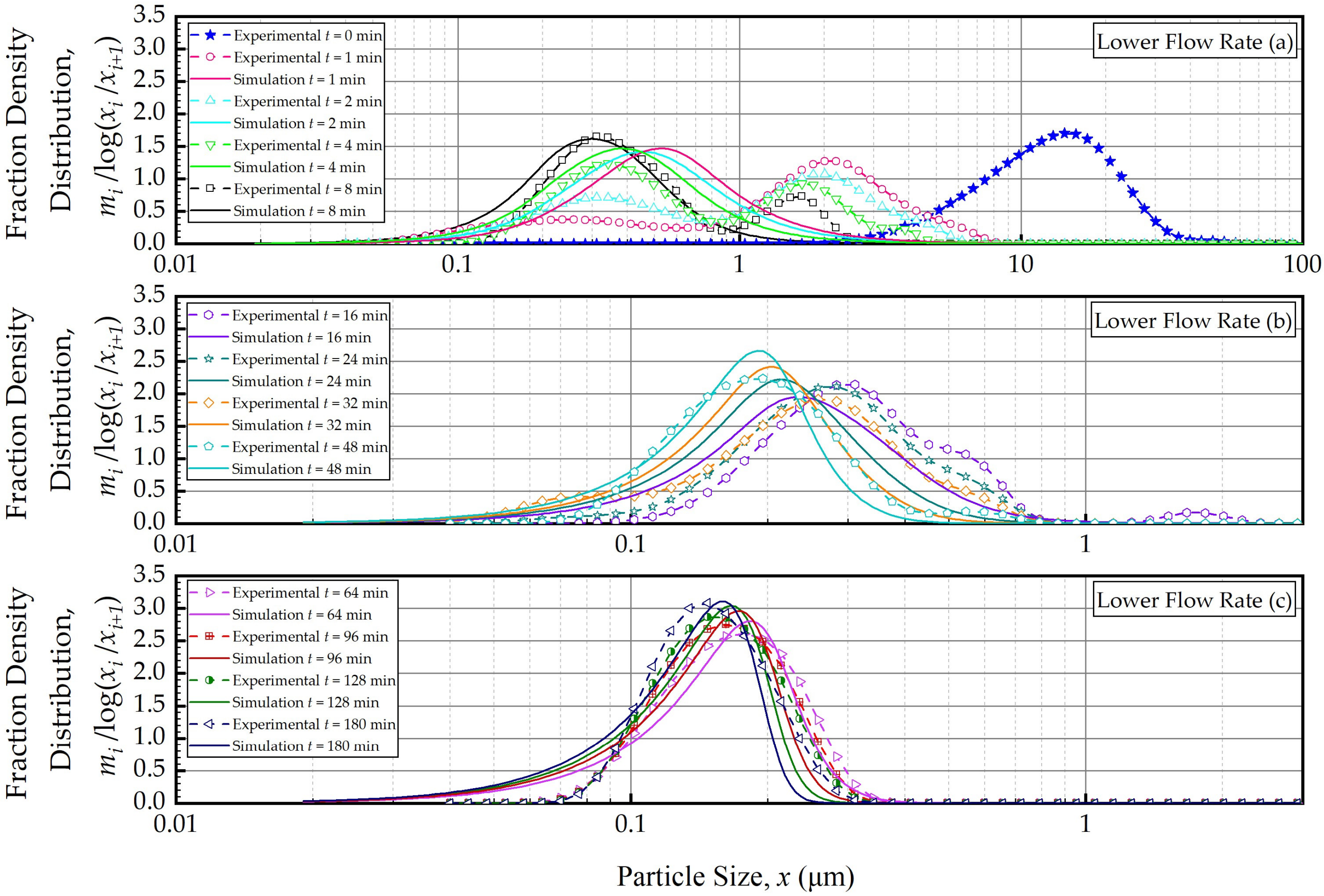

Figure S4. Temporal variation of the mass fraction density distribution during (a) the first 8 min of milling, (b) between 16 and 48 min of milling, and (c) thereafter for Run 4: volumetric flow rate of 63 mL/min and batch volume of 236 mL. Simulation: PBM with Model C and its fitted parameters.

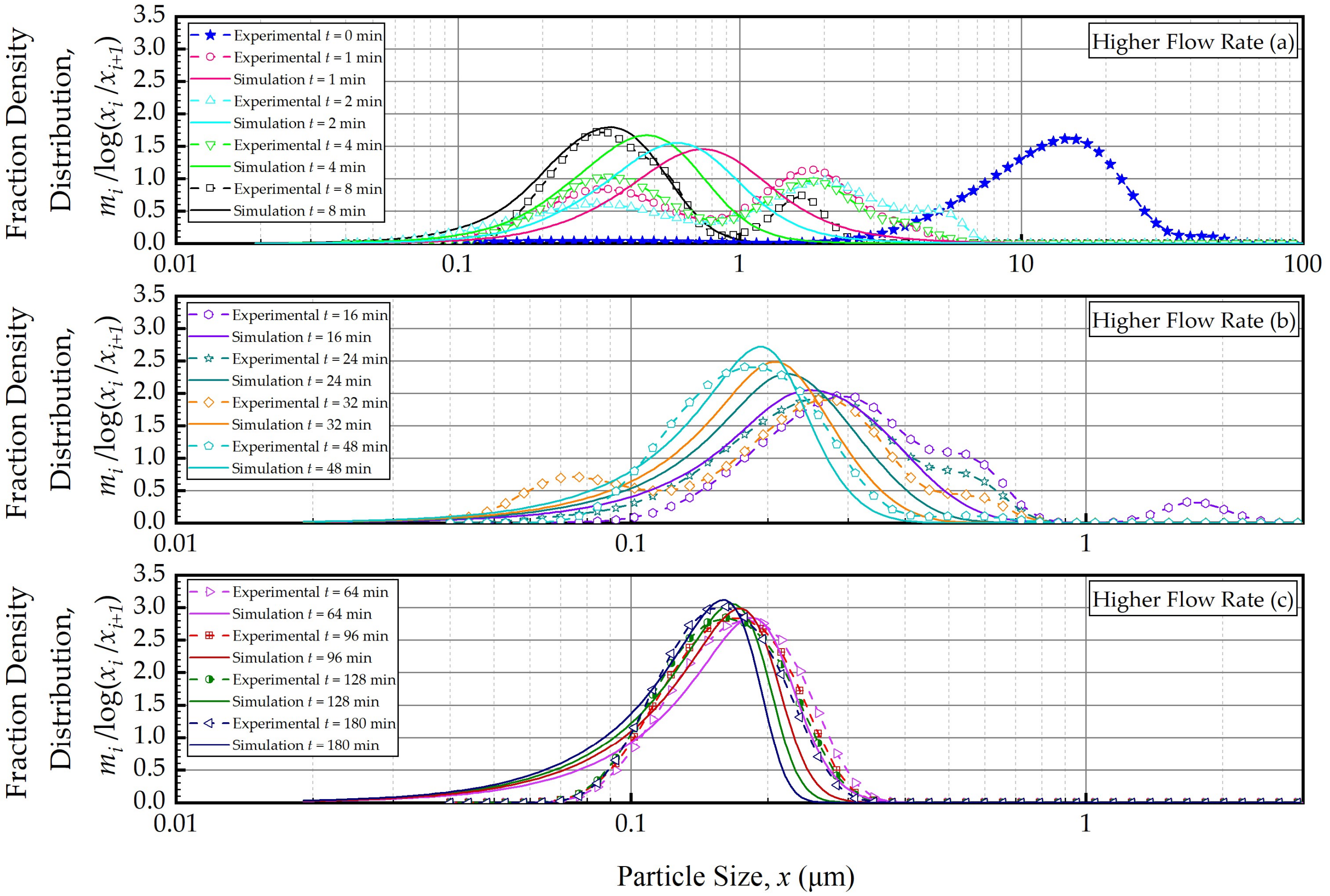

Figure S5. Temporal variation of the mass fraction density distribution during (a) the first 8 min of milling, (b) between 16 and 48 min of milling, and (c) thereafter for Run 5: volumetric flow rate of 250 mL/min and batch volume of 236 mL. Simulation: PBM with Model C and its fitted parameters.

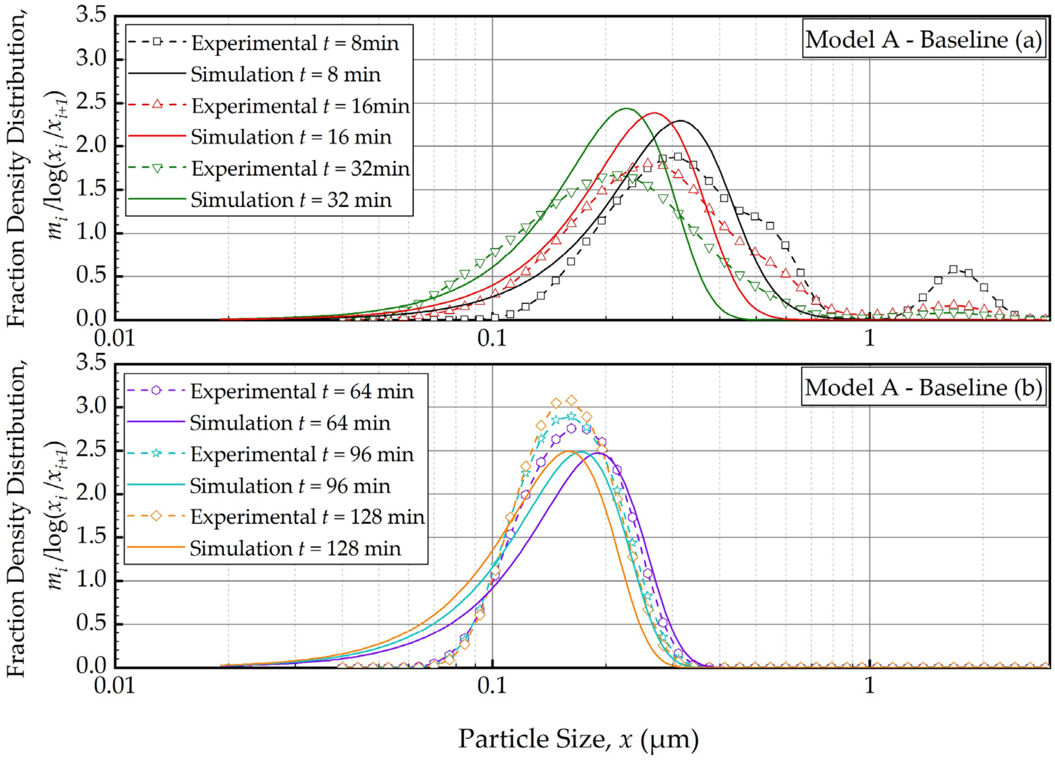

Figure S6. Temporal variation of the mass fraction density distribution during (a) the first 32 min of milling and (b) thereafter for the baseline experiment: volumetric flow rate of 126 mL/min and batch volume of 236 mL (Run 1). Simulation: PBM with Model A and its fitted parameters.

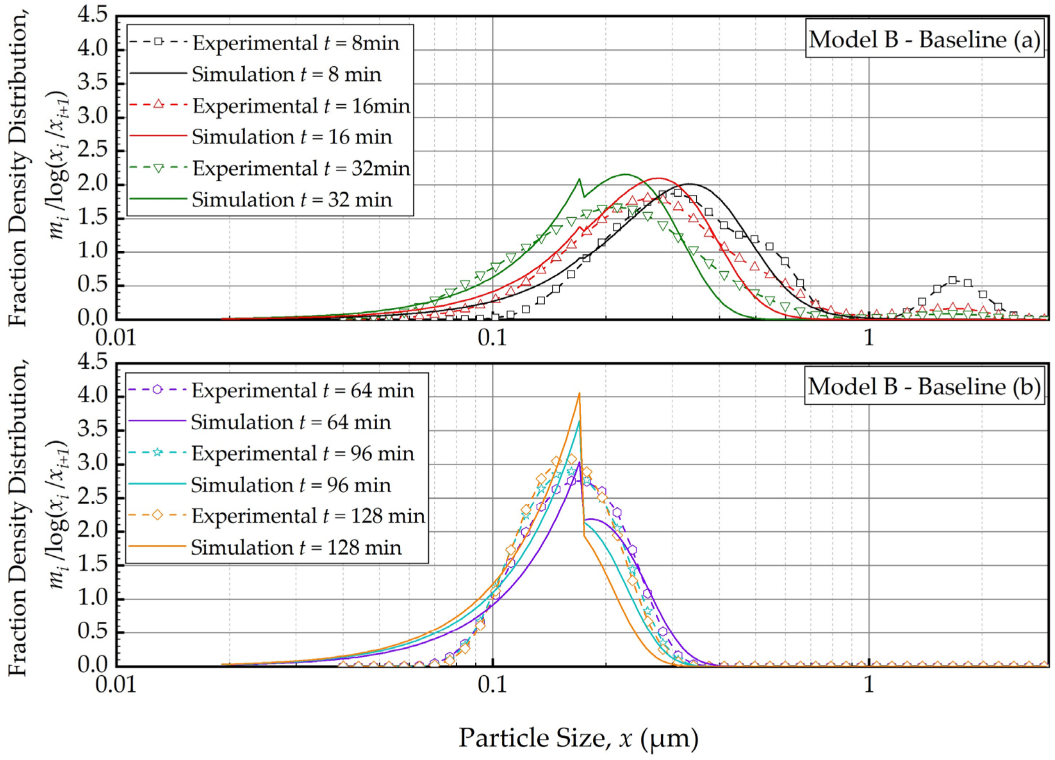

Figure S7. Temporal variation of the mass fraction density distribution during (a) the first 32 min of milling and (b) thereafter for the baseline experiment: volumetric flow rate of 126 mL/min and batch volume of 236 mL (Run 1). Simulation: PBM with Model B and its fitted parameters.

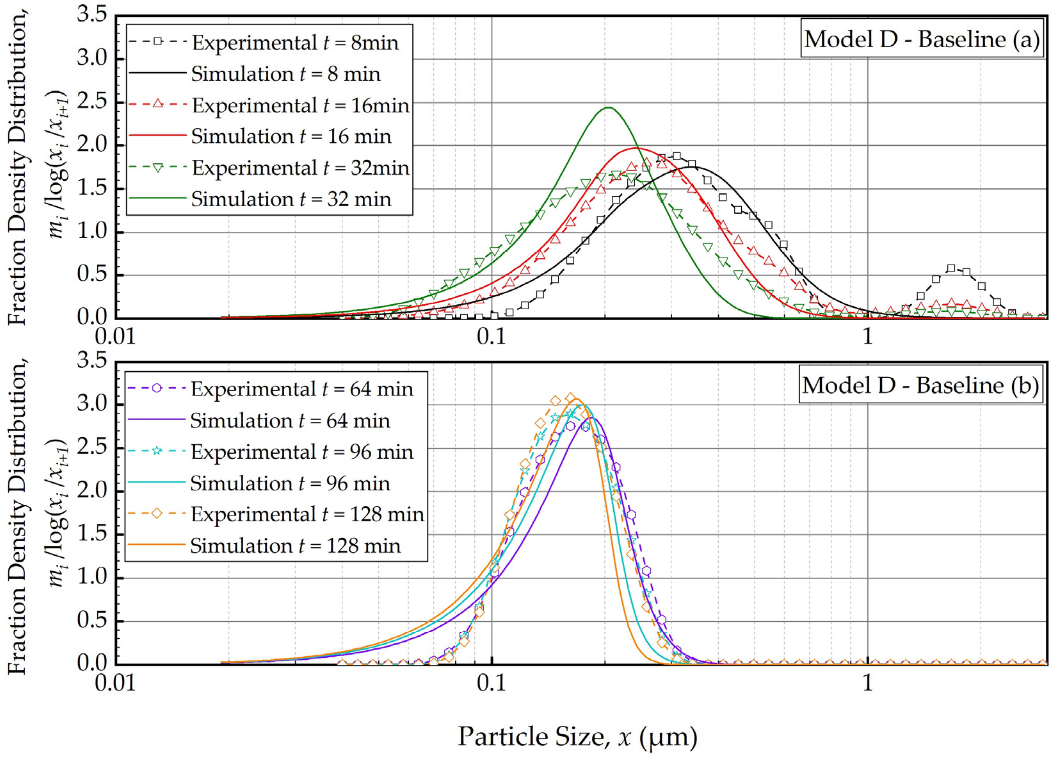

Figure S8. Temporal variation of the mass fraction density distribution during (a) the first 32 min of milling and (b) thereafter for the baseline experiment: volumetric flow rate of 126 mL/min and batch volume of 236 mL (Run 1). Simulation: PBM with Model D and its fitted parameters.

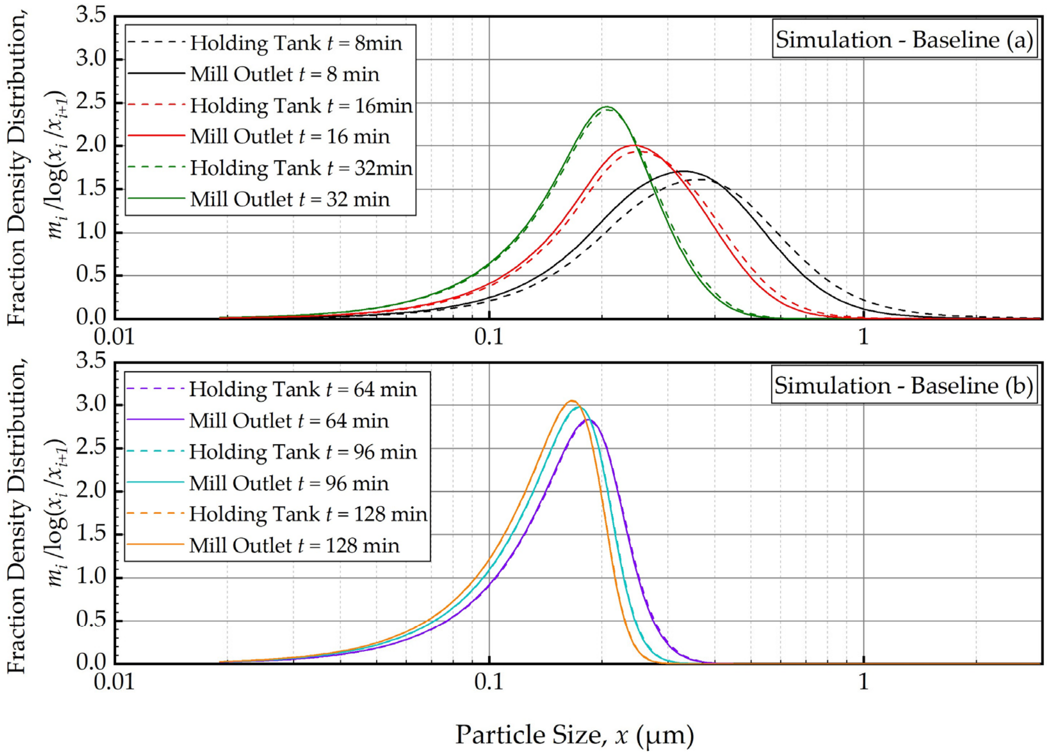

Figure S9. Simulated temporal variation of the mass fraction density distribution in the milling chamber and the holding tank during (a) the first 32 min of milling and (b) thereafter for the baseline experiment: volumetric flow rate of 126 mL/min and batch volume of 236 mL (Run 1, Model C).
